# Superstructural phase transitions in polymer-grafted nanooctahedra

**DOI:** 10.1126/sciadv.adw2740

**Published:** 2025-07-18

**Authors:** Baixu Zhu, Jun Chen, Ruipeng Li, Jarett Ren, Yi Wang, Yaxu Zhong, Yang Liu, Akira Yasuhara, Mayu Kakefuda, Yoshitaka Aoyama, Thi Vo, Xingchen Ye

**Affiliations:** ^1^ Department of Chemistry, Indiana University, Bloomington, IN 47405, USA.; ^2^ National Synchrotron Light Source II, Brookhaven National Laboratory, Upton, NY 11973, USA.; ^3^ Department of Chemical and Biomolecular Engineering, Johns Hopkins University, Baltimore, MD 21218, USA.; ^4^ JEOL Ltd., 3-1-2 Musashino, Akishima, Tokyo 196-8558, Japan.

## Abstract

Superlattices of polyhedral nanocrystals exhibit emergent properties defined by their structural arrangements, but native nanocrystal ligands often limit their programmability. Polymeric ligands address this limitation by enabling tunable nanocrystal softness through modifications of polymer molecular weight and grafting density. Here, we investigate phase transitions in polymer-grafted nanooctahedra by varying polymer length, nanocrystal size, truncation, and ligand density. In two-dimensional superlattices, longer polymers or smaller nanooctahedra induce a transition from orientationally ordered to hexagonal rotator lattices. In three-dimensional superlattices, increasing polymer length drives transitions from Minkowski to body-centered cubic and plastic hexagonal close-packed phases, while higher grafting densities further enable transitions to simple hexagonal phases. Polymer brush and thermodynamic perturbation theories, supported by Monte Carlo simulations, uncover the entropic and enthalpic forces that govern these transitions. This work highlights the versatility of polymer-grafted anisotropic nanocrystals as building blocks for designing hierarchical superstructures and metamaterials with customizable properties.

## Teaser

Polymer ligands drive structural transitions in polyhedral nanocrystal superlattices, opening avenues for materials design.

## INTRODUCTION

In recent years, substantial advances in colloidal nanochemistry have established nanocrystal (NC) shape as a powerful tool for directing hierarchical mesoscale organization. Among these, polyhedral NCs have attracted substantial attention as model systems for studying how particle shape and interparticle interactions govern assembly thermodynamics and kinetic pathways (*1*–*15*). Of particular interest is the ability of polyhedral NCs to harness the delicate balance between enthalpic and entropic interactions, enabling precise control over their self-assembled superstructures (*8*, *16*–*23*). For example, hard (noninteracting) NCs use entropy maximization to drive facet-to-facet alignment, favoring the formation of close-packed structures, as predicted by both theoretical and simulation studies (*24*–*28*). In contrast, soft NCs, those functionalized with ligand coatings, preferentially align along edges and vertices because of ligand-mediated enthalpic interactions (*29*–*31*). These configurations disrupt the preprogrammed directional interactions dictated by the NC core geometry, enabling the formation of open lattice structures. On a fundamental level, these findings highlight the promise of changing NC softness to create versatile nanoscale building blocks capable of assembling into diverse superstructures. From an application standpoint, the tunable orientational ordering offers precise control over the emergent properties of NC-based metamaterials. For example, controlling the orientational alignment of high-curvature regions (e.g., vertices or edges) in metallic NCs can significantly enhance local electric fields, thus benefiting applications such as chemical sensing (*32*–*36*) and plasmon-mediated catalysis (*37*).

However, control over NC softness is often constrained by the limited selection of ligands compatible with size and shape control during synthesis. As a result, as-synthesized polyhedral NCs typically fall into one of two categories: (i) hard-like particles that form densely packed superstructures (*7*, *38*–*40*) or (ii) soft particles that produce low–packing density superstructures (*30*, *31*, *41*–*43*). Moreover, widely studied noble-metal NC cores possess intrinsically high Hamaker constants that enhance van der Waals interactions between NCs, thereby promoting face-to-face alignment and dense packings (*33*). Such preferences for close-packed morphologies often diminish or obscure the role that NC surface ligands play on self-assembly. Consequently, experimental realization of polyhedral NCs with a broad spectrum of particle softness, along with a deeper understanding of their role in directing hierarchical assemblies, remains a significant and open challenge.

Macromolecular ligand engineering offers a robust strategy for creating polyhedral NCs with tunable softness. Seminal works using DNA-based ligands have shown precision control over softness and specific, directional interactions between NCs (*44*). However, such systems often exhibit limited thermal stability because of the narrow melting temperature range of DNA. Polymeric ligands offer key advantages such as facile synthesis, chemical versatility, and enhanced thermal stability over a broad temperature range. In addition, the inherent flexibility of polymers enables the formation of deformable ligand shells around NCs, mitigating assembly frustrations arising from local defects and particle polydispersity. Previous works have shown that precision control over polymer molecular weight and grafting density enables tunable softness in spherical NCs (*16*, *18*, *21*, *45*–*54*), which drives phase transitions during self-assembly, such as face-centered cubic to body-centered cubic (BCC) transformation (*17*, *55*, *56*). Experimental studies have also revealed phase transitions in polymer-grafted nonspherical NCs, such as triangular nanoprisms (*16*) and nanotetrahedra (*8*), although these transitions have so far been limited to two-dimensional (2D) superstructures. Systematic control over 3D assembly behaviors of polymer-grafted polyhedral NCs remains largely unexplored.

Here, we developed a synthesis method for size-tunable core/shell MnO@Mn_3_O_4_ octahedral NCs (hereafter referred to as nanooctahedra) and studied the self-assembly phase behavior of polystyrene (PS)–grafted MnO@Mn_3_O_4_ nanooctahedra. Phase transitions in both 2D and 3D superlattices were systematically explored by varying the nanooctahedron edge length, polymer ligand length, and grafting density. In 2D superlattices, increasing NC softness triggered a transition from a hexagonal lattice with orientational order to a hexagonal rotator structure. For 3D superlattices, tuning NC softness facilitated the formation of four distinct superstructures with high phase purity: Minkowski, BCC, plastic hexagonally close-packed (pHCP), and simple hexagonal (SH). This work represents a comprehensive demonstration of how precision control over non–DNA-based polymer functionalization can guide the assembly of polyhedral NCs into a full spectrum of superlattice symmetries with high phase purity (*57*). The observed rich phase behaviors were further rationalized using polymer brush theory and thermodynamic perturbation theory, providing a microscopic understanding of the driving forces governing these superstructural phase transitions.

## RESULTS

### Synthesis and characterization of PS-grafted MnO@Mn_3_O_4_ nanooctahedra

**Fig. 1.**
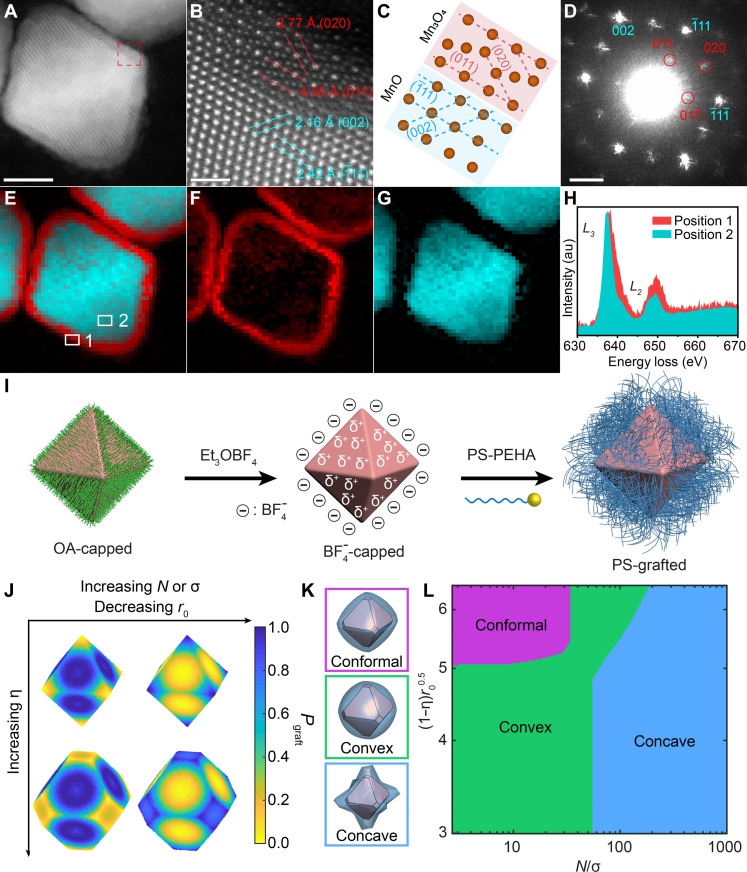
Synthesis, characterization, and ligand conformation analysis of PS-grafted MnO@Mn_3_O_4_ nanooctahedra. (**A** to **D**) HAADF-STEM image (A), atomic-resolution HAADF-STEM image (B), the corresponding crystal-structure model illustrating the core/shell interface (C), and WAED pattern (D) of single ${\text{BF}}_{4}^{-}$-capped MnO@Mn_3_O_4_ nanooctahedron. The lattice planes of MnO and Mn_3_O_4_ are highlighted in blue and red, respectively. In (C), oxygen atoms are omitted for clarity. (**E** to **H**) Manganese oxidation state mapping of single MnO@Mn_3_O_4_ nanooctahedron. EELS maps of the MnO phase (F), the Mn_3_O_4_ phase (G), and their overlay (E). (H) EELS spectra acquired from positions 1 and 2, as marked in (E). a.u., arbitrary unit. (**I**) Schematic illustration of the two-step ligand-exchange process. (**J**) Spatial distribution of ligand densities on a nanooctahedron as a function of nanooctahedron edge length ($r_{o}$), degree of truncation (η), polymer ligand chain length (*N*), and grafting density (σ). (**K**) Schematic illustration of distinct equilibrium corona shapes extending from the nanooctahedron core. (**L**) Theoretical diagram illustrating the equilibrium corona shape around the nanooctahedron core as a function of key parameters. Scale bars, 10 nm (A), 1 nm (B), and 2 nm^−1^ (D).

MnO nanooctahedra in the rock-salt phase were synthesized via the thermal decomposition of manganese oleate, formed in situ by reacting manganese acetate with oleic acid (OA) (Materials and Methods and fig. S2, A and B) (*58*). The octahedral morphology was clearly visualized in the 3D model obtained through transmission electron microscopy (TEM) tomography reconstruction (Materials and Methods and movie S1). The edge lengths of the nanooctahedra were tunable between 28 and 50 nm by adjusting the reaction temperature. The degree of truncation (η) decreased with increasing edge length, measured as η = 0.29 for 28-nm, η = 0.18 for 40-nm, and η = 0.17 for 50-nm nanooctahedra (fig. S2, C to E). High-angle annular dark-field scanning TEM (HAADF-STEM) images of edge-up nanooctahedra confirmed their core-shell structure (Fig. 1, A and B). The nanobeam electron diffraction (NBED) pattern of a single nanooctahedron revealed two distinct diffraction patterns corresponding to the [110] zone axis of the cubic rock-salt MnO core and the [100] zone axis of the tetragonal spinel Mn_3_O_4_ shell, formed through surface oxidation (Fig. 1D). Atomic-resolution HAADF-STEM imaging further indicated that the (011) planes of the Mn_3_O_4_ shell and the (111) planes of the MnO core were epitaxially related, aligned parallel to the exposed facets of the nanooctahedra (Fig. 1, B and C). In addition, electron energy loss spectroscopy (EELS) mapping (Fig. 1, E to H) confirmed the valence states of manganese in the core and shell regions as Mn(II)O and Mn(II, III)_3_O_4_, respectively, through analysis of the Mn *L*_*3*_/*L*_*2*_ white-line intensity ratios (Fig. 1, E to H and the Supplementary Materials) (*59*, *60*).

Bromine-terminated PS (PS-Br) was synthesized using activators regenerated by electron transfer–atom transfer radical polymerization (*61*), followed by terminal group conversion to produce pentaethylenehexamine-terminated PS (PS-PEHA) ligands (Materials and Methods and and table S1) (*17*). The molecular weight of PS-Br was characterized via gel permeation chromatography (GPC) (fig. S3). Functionalization of MnO@Mn_3_O_4_ nanooctahedra with PS-PEHA ligands was achieved using a two-step ligand-exchange method (Fig. 1I) (*16*). In the first step, triethyloxonium tetrafluoroborate (Et_3_OBF_4_) was used to remove the native OA ligands via an alkylation reaction (*62*). The near-complete removal of OA was confirmed by the significantly diminished intensity of C-H stretching vibrations in the range of 3000 to 2800 cm^−1^, as observed in Fourier transform infrared (FTIR) spectra (fig. S4; detailed descriptions can be found in the Supplementary Materials). In the second step, PS-PEHA ligands were grafted onto the ligand-stripped NCs, with the grafting density precisely controlled by the feeding ratio. Successful grafting of PS-PEHA ligands was verified by the appearance of aromatic C-H stretching vibrations, N-H bending, and in-plane aromatic C-H bending vibrations in the FTIR spectra, along with a solubility shift of the NCs from alkanes to toluene (*16*). The ligand grafting density was further quantified through thermogravimetric analysis (TGA) (Materials and Methods, fig. S5, and table S2).

### Theory prediction of polymer ligand distribution on nanooctahedron surfaces

To gain a fundamental understanding of how macromolecular ligand engineering modulates NC softness, we used the polymer brush scaling theory to predict the spatial distribution of grafted PS ligands as a function of key experimental parameters: grafting density, polymer molecular weight, nanooctahedron truncation, and edge length. Briefly, our theory extends the star polymer scaling theory (*63*) by explicitly accounting for the influence of local curvature variations on the entropic penalty associated with chain confinement (*20*, *64*–*66*). For PS-grafted NCs dispersed in a good solvent, the end-to-end distance R of an end-functionalized polymer chain is expressed as R∼roσ1/5ν1/5b2/5[NbΩri]3/5, where $r_{o}$ is the NC insphere radius, σ is the grafting density, N is the polymer chain length in terms of the number of monomers, ν is the excluded volume for a monomer with Kuhn length b, and Ω represents the surface position on the NC, defined as a function of the azimuthal and polar angles. The Flory energy is then defined as βF~R2Nb2+ν N2f(ΩR)3, which was used to compute the Boltzmann-weighted probability of chain grafting ($P_{\text{graft}}∼e^{-\beta F}$) at different surface locations (Ω) on the NC (Fig. 1J). Monte Carlo grafting simulations were performed to graft polymer chains at various positions on the NC surface, using the standard Metropolis criterion with a grafting probability defined by $P_{\text{graft}}$ (*67*). Averaging over 10,000 independent Monte Carlo grafting simulations provided a thermodynamic average of the equilibrium corona shape (the shape of the polymer shell) surrounding the nanooctahedron core (Fig. 1K). Analysis of these results revealed three distinct corona morphologies accessible within the phase space defined by grafting density, polymer length, and nanooctahedron edge length/truncation. These corona morphologies were classified as conformal, convex, and concave (Fig. 1L). To illustrate theoretical predictions across four distinct parameters in a single 2D diagram, we selected composite parameters $\left(1-\eta \right)r_{o}^{1/2}$ and $N\sigma^{-1}$ as the *x* and *y* axes, respectively.

Because all grafted chains are chemically identical and immersed in good solvent during synthesis, interpolymer interactions are effectively screened, leaving only steric repulsions between neighboring monomers. Thus, transitions in corona morphology are entropy-driven and governed by chain packing on the NC surface. Conformal coronas arise under conditions of low grafting density, short chain lengths, large edge lengths, and low truncations. Within this regime, the polymer chains are too short relative to the NC core size. The entropy gained by partitioning chains to high-curvature regions (to reduce chain extension) is insufficient to offset the configurational entropy loss required to maintain an isotropic surface distribution. Consequently, the lowest-energy configuration involves polymer chains forming short, uniform brushes that conform to the intrinsic shape of the NC core, producing the conformal corona. This behavior is analogous to the Alexander–de Gennes brushes observed in planar grafting (*68*, *69*). As chain length and/or grafting density increase, chain confinement intensifies, driving a transition to either the convex or concave corona regimes. For convex coronas, the entropic cost of increased confinement remains low enough that chain partitioning to high-curvature regions is still unfavorable. More specifically, the entropy-mediated confinement energy is comparable to the available thermal energy within the surrounding environment, allowing for thermal fluctuations to locally resolve confinement between neighboring chains. As a result, chains stretch in place rather than undergo bulk surface reorganization. Since large facets on NC surfaces experience greater confinement because of the smaller solid angle available for chain packing, face-anchored chains extend further relative to those at corners or edges, transforming the planar morphology into a spherical cap. This cap-like corona motif at NC facets defines the convex corona morphology. With further increases in chain length and/or grafting density, the conformational entropy gained by chain relaxation at edges and corners dominates. This significant entropic gain drives preferential partitioning of chains to edges and corners while depleting grafts from NC faces, forming the protruded edge and corner motifs characteristic of concave coronas. Similarly, decreasing the nanooctahedron edge length increases the degree of chain confinement, which drives the same systematic shifts from conformal to convex to concave corona morphologies.

Through the lens of chain entropy, we can provide additional physical interpretations for the composite parameters $\left(1-\eta \right)r_{o}^{1/2}$ and $N\sigma^{-1}$. The term $\left(1-\eta \right)r_{o}^{1/2}$, governed by NC core geometry, serves as a proxy for the available surface area for polymer functionalization on the nanooctahedron. In contrast, $N\sigma^{-1}$ reflects polymer-specific contributions, capturing entropic changes associated with chain extension. At a fixed grafting density (σ), lower $N\sigma^{-1}$ values correspond to shorter chains, which incur a smaller entropic penalty upon extension. When paired with a high $\left(1-\eta \right)r_{o}^{1/2}$ (i.e., large surface area), this results in a minimal overall entropic cost, favoring a conformal corona morphology. As chain length increases (higher $N\sigma^{-1}$), the entropic cost of chain extension rises. If coupled with a lower $\left(1-\eta \right)r_{o}^{1/2}$ (reduced surface area), this amplifies the entropic penalty for uniform grafting, driving chains to preferentially localize at high-curvature sites, thereby promoting a concave corona morphology. Similarly, increasing grafting density at constant chain length also raises $N\sigma^{-1}$, enhancing chain extension and reinforcing the entropy-driven transition from conformal to concave morphologies.

### 2D superlattices assembled from PS-grafted MnO@Mn_3_O_4_ nanooctahedra

**Fig. 2.**
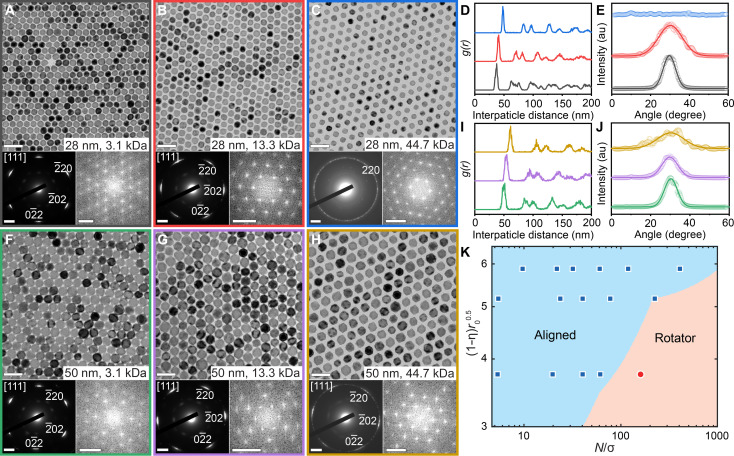
Structural transition in 2D superlattices of PS-grafted MnO@Mn_3_O_4_ nanooctahedra. (**A** to **C** and **F** to **H**) TEM images (top), the corresponding WAED patterns (bottom left), and FFT patterns (bottom right) of 2D superlattices assembled from 28 nm (A to C) and 50 nm (F to H) MnO@Mn_3_O_4_ nanooctahedra functionalized with various PS-PEHA ligands. Scale bars, 100 nm for TEM images, 2 nm^−1^ for WAED patterns, and 0.05 nm^−1^ for FFT patterns. (**D** and **I**) Radial distribution function g(r) of various 2D superlattices calculated from the corresponding TEM images. (**E** and **J**) Radial intensity profiles obtained by radially integrating the {220}_MnO_ diffraction intensities from the WAED patterns and the corresponding Gaussian fits. (**K**) Phase diagram of 2D polymer-grafted nanooctahedron superlattices as a function of key parameters obtained from Monte Carlo simulations, overlaid with experimental data points. The diagram distinguishes superlattice phases with orientational order of the NC cores (“Aligned”) from those without orientational order (“Rotator”). The color of the solid background represents the theoretically predicted equilibrium structure using ALT.

2D superlattices of PS-grafted MnO@Mn_3_O_4_ nanooctahedra were fabricated by slow evaporation of a toluene solution of NCs on a diethylene glycol (DEG) surface (*70*). TEM images and their corresponding fast Fourier transform (FFT) patterns revealed that the superlattices exhibited sixfold hexagonal symmetry across nanooctahedron edge lengths ranging from 28 to 50 nm and PS-PEHA molecular weights between 3.1 and 44.7 kDa (Fig. 2 and figs. S6 to S8). Sharp peaks in the radial distribution function g(r), calculated from the TEM images, further confirmed the long-range translational order within the 2D superlattices (Fig. 2, D and I, and fig. S10, A to C). The ratios of the first three peak positions in g(r) (1:1.73:2) were consistent with the hexagonal symmetry of the 2D lattices.

Wide-angle selected-area electron diffraction (WAED) patterns exhibited six sharp {220} diffraction spots of MnO, indicating that the nanooctahedra were orientationally ordered, with the MnO [111] zone axis perpendicular to the superlattice plane and the nanooctahedron facets aligned parallel to the plane (Fig. 2 and fig. S9A). As the PS-PEHA molecular weight increased, the six {220}_MnO_ diffraction spots broadened and transitioned into six diffraction arcs in the WAED patterns. This broadening corresponded to an increase in the full width at half maximum of the peaks in the radial intensity profiles (Fig. 2, E and J, and fig. S10, D to F), suggesting a reduction in the orientational order of the nanooctahedra.

The orientational order of MnO@Mn_3_O_4_ nanooctahedra within 2D superlattices depended not only on the PS-PEHA molecular weight but also on the nanooctahedron edge length. At a fixed PS-PEHA molecular weight, the orientational order decreased as the NC size decreased (fig. S11A), suggesting that NC softness (the ratio of polymer ligand length to NC size) plays a critical role (*8*, *16*). Both increasing the PS-PEHA molecular weight and decreasing the NC size led to an increase in NC softness, which, in turn, reduced the orientational order of the NCs in the 2D superlattices. When the smallest nanooctahedra (28-nm edge length) were grafted with the longest PS-PEHA ligands examined (44.7 kDa), the WAED pattern exhibited a diffraction ring with nearly uniform radial intensity, indicating the formation of a plastic hexagonal phase characterized by random orientational ordering. Similar 2D plastic hexagonal phases have been observed in superlattices assembled from polymer-grafted NCs, including nanotetrahedra (*8*) and triangular nanoprisms (*16*), when grafted with polymers of sufficiently high molecular weight. This behavior appears to be a general phenomenon in the 2D assembly of polymer-grafted NCs.

Theoretical calculations combining corona shape predictions and associating lattice thermodynamic perturbation theory (ALT) (*20*, *65*, *71*) for 2D lattice configurations revealed trends in orientational ordering consistent with experimental observations. To unify theoretical predictions and experimental data across four different parameter sets into a single phase diagram, we used the same set of composite parameters of $\left(1-\eta \right)r_{o}^{1/2}$ and $N\sigma^{-1}$ for the *x* and *y* axes, respectively, used in the corona morphology phase diagram (Fig. 1L). Experimental data points were overlaid on top of theoretical predictions for direct comparison (Fig. 2K). The analysis revealed that orientationally aligned configurations were favored at low polymer chain lengths and higher grafting densities (low $N\sigma^{-1}$). In this regime, the NC coronas typically adopt conformal or convex morphologies that closely mirror the geometry of the underlying core. This leads to a synergistic interplay between shape-driven directional entropic forces (*72*, *73*) and polymer-mediated interparticle interactions, maximizing facet alignment among NCs. This preference for facet alignment resulted in strong face-to-face inter-NC attractions, driving the formation of an orientationally aligned hexagonal 2D lattice, as observed experimentally. At higher chain lengths and grafting densities, the thermodynamically favored corona morphologies shifted to those exhibiting concave geometries. Correspondingly, theory predicted an analogous shift to a rotator hexagonal phase. In this regime, the directional interactions imposed by the grafted polymer chains diverged from those preferred by the NC core geometry. Specifically, polymer-mediated interactions favored corner/edge alignments, while core-mediated interactions preferred face alignments. This mismatch between competing forces disrupted orientational ordering within the self-assembled lattice. The resulting 2D rotator phase, characterized by the absence of preferred orientational alignment between NCs, reflected an effective “canceling out” between polymer-mediated and core-mediated interactions.

To further confirm that changes in polymer distribution on the NC surface (corona morphology) drive the observed phase transitions, we measured the average face-to-face distances between adjacent nanooctahedra in the hexagonal lattice (fig. S11B). We then normalized these distances using the scaling relation ${M_{n}}^{0.5}\times \sigma^{0.25}$ to eliminate the effects of varying molecular weight ($M_{n}$) and grafting density (σ) of PS-PEHA ligands (fig. S12) (*63*). Our measurements revealed that the normalized face-to-face distance decreased with increasing polymer chain length (table S3), indicating fewer polymers present at the NC faces. This reduction corresponds to a transition from conformal/convex to concave corona morphologies, consistent with theoretical predictions. Last, we note that aligned phases were observed in both theory predictions and experiments near the boundary between concave and convex corona morphologies. Specifically, these phases assembled in regions of larger NC core sizes and lower polymer grafting densities, even when individual functionalized NCs exhibited concave corona morphologies. The combination of larger NC core size and reduced grafting density enhanced entropic effects because of an increased contribution of core-mediated interactions. Consequently, unlike in the rotator phase limit, entropic forces were strong enough to overcome polymer-mediated corner and edge alignments, preserving orientationally ordered NC assemblies. These findings highlight the importance of capturing the interplay between enthalpic and entropic interactions in directing self-assembly and suggest that such counterbalancing effects can be leveraged to assess a wider variety of NC superlattices.

### 3D superlattices assembled from PS-grafted MnO@Mn_3_O_4_ nanooctahedra

**Fig. 3.**
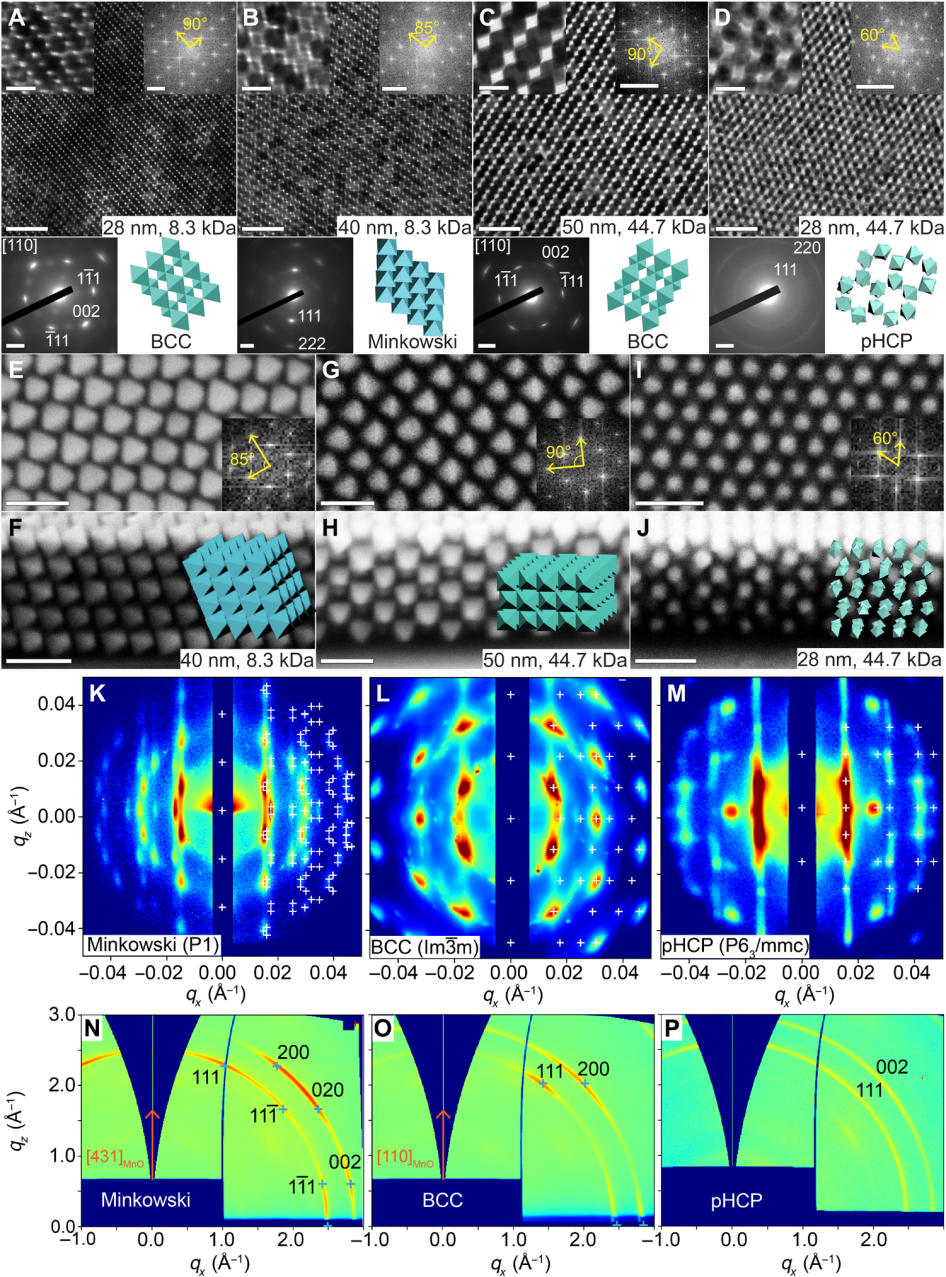
Phase transition in 3D superlattices of PS-grafted MnO@Mn_3_O_4_ nanooctahedra. (**A** to **D**) Characterization of four distinct types of 3D superlattices assembled from PS-grafted MnO@Mn_3_O_4_ nanooctahedra. Each panel shows a low-magnification TEM image (top), a high-magnification TEM image (top-left inset), the corresponding FFT pattern (top-right inset), the WAED pattern (bottom-left inset), and a structural model (bottom-right inset). Scale bars, 200 nm for low-magnification TEM images, 50 nm for high-magnification TEM images, 2 nm^−1^ for WAED patterns, and 0.05 nm^−1^ for FFT patterns. (**E** to **J**) Top-view SEM images (E, G, and I) with the corresponding FFT patterns (insets) and cross-sectional SEM images (F, H, and J) for the Minkowski (E and F), BCC (G and H), and pHCP (I and J) superlattices. Scale bars, 100 nm (E to J). (**K** to **P**) GTSAXS (K to M) and GIWAXS (N to P) patterns of the Minkowski (K and N), BCC (I and O), and pHCP (M and P) superlattices, corresponding to the structures shown in (B), (A), and (D), respectively.

To further investigate how tuning NC softness influences self-assembly, we formed 3D superlattices by increasing the amount of NCs in the drop-cast solution by approximately tenfold. The evaporation rate of toluene is controlled as approximately 5 μl/min, allowing the toluene solution to evaporate over roughly 40 hours. This deliberate control over the evaporation process facilitates the formation of thermodynamically favored structures, effectively minimizing the impact of solvent evaporation on the assembly process. MnO@Mn_3_O_4_ nanooctahedra with edge lengths of 28 and 40 nm, grafted with 8.3-kDa PS-PEHA ligands, assembled into distinct superstructures (Fig. 3, A and B, and figs. S13 to S16). The FFT pattern of the 28-nm nanooctahedron superlattice displayed two orthogonal basic reciprocal vectors with a length ratio of 1.41, closely matching the simulated FFT pattern of the [110] projection for a BCC structure (Fig. 3A and fig. S13F). The corresponding WAED pattern confirmed orientational order, with the (110)_MnO_ plane of the nanooctahedra parallel to the (110)_SL_ plane of the BCC superlattice and the [001]_MnO_ direction aligned with the [001]_SL_ direction (Fig. 3A). In contrast, the FFT pattern of the 40-nm nanooctahedron superlattice showed two nonorthogonal basic reciprocal vectors with an angle of 85° and a length ratio of 1.45 (Fig. 3B and fig. S15F). The corresponding WAED pattern exhibited only one set of {111}_MnO_ diffraction spots. These findings indicate that the 40-nm nanooctahedra assembled into a Minkowski lattice with triclinic symmetry (*74*), where the (001)_SL_ plane was parallel to the substrate.

Cross-sectional SEM images further revealed distinct NC arrangements between the two superlattices. In the cleaved (001)_SL_ plane of the BCC superlattice formed by 50-nm nanooctahedra grafted with 44.7-kDa PS-PEHA ligands, the individual nanooctahedra were oriented edge-up relative to the substrate and exhibited vertex-to-vertex contact with neighboring NCs (Fig. 3, G and H) (*30*). The observed ABAB stacking of nanooctahedra parallel to the substrate was consistent with the ABAB stacking of the (110)_SL_ plane in the BCC superlattice. Conversely, the cross-sectional SEM image of the Minkowski lattice formed by 40-nm nanooctahedra grafted with 8.3-kDa PS-PEHA ligands showed the (010) plane of the Minkowski lattice. In this structure, all nanooctahedra were identically oriented and made contact with neighboring NCs through partial face-to-face overlap (Fig. 3, E and F) (*40*). The lack of twofold symmetry in the Minkowski (001) plane was consistent with the triclinic lattice symmetry. A third type of superlattice was observed with 28-nm nanooctahedra grafted with 44.7-kDa PS-PEHA ligands. The FFT pattern of the TEM image exhibited hexagonal symmetry, while the WAED pattern showed diffraction rings indicative of the absence of NC orientational order (Fig. 3D and fig. S17). Cross-sectional SEM images revealed ABAB stacking parallel to the substrate (Fig. 3J). Together, these results indicate that the nanooctahedra formed a pHCP structure.

The superlattice structures and orientations were further analyzed using grazing-incidence transmission small-angle x-ray scattering (GTSAXS) and grazing-incidence wide-angle x-ray scattering (GIWAXS) techniques. The transmission scattering signals were preserved without distortion under the grazing incidence geometry. To enhance transmission signals and avoid overlap between transmission and reflection signals in the low-*q* range, we used a relatively large incident angle of 0.5°. Indexing of the GTSAXS/GIWAXS patterns revealed the superlattice structure and NC orientational order in both in-plane and out-of-plane directions. The Minkowski structure, assembled from 8.3-kDa PS-grafted 40-nm nanooctahedra, exhibited [112]_SL_ and [431]_MnO_ orientations perpendicular to the substrate, with lattice constants *a* = 42.0 nm and *b* = *c* = 45.36 nm (Fig. 3, K and N, and fig. S18). The BCC superlattice, assembled from 8.3-kDa PS-grafted 28-nm nanooctahedra, displayed [110]_SL_ and [110]_MnO_ orientations perpendicular to the substrate, with a lattice constant of *a* = 37.0 nm (Fig. 3, L and O, and fig. S19). The pHCP superlattice, assembled from 44.7-kDa PS-grafted 28-nm nanooctahedra, had [001]_SL_ orientation perpendicular to the substrate, with lattice constants *a* = *b* = 48.0 nm and *c* = 78.2 nm (Fig. 3, M and P, and fig. S20). Random NC orientations were evidenced by the diffuse rings corresponding to the (111)_MnO_ and (200)_MnO_ peaks in the GIWAXS pattern. Lattice contraction perpendicular to the substrate was observed in all three superlattices (fig. S21). This vertical lattice shrinkage, attributed to anisotropic solvent evaporation during the final stages of superlattice formation, has been widely reported in various soft-matter systems (*17*, *75*–*77*). The relatively low *z*-axis shrinkage ratio (1%) in the Minkowski phase was due to the denser packing of NCs. In addition, superlattice contraction caused slight NC rotations. In the Minkowski structure, individual nanooctahedra rotated 9.5° around the [$\bar{1}11$]_MnO_ axis, shifting the NC zone axis perpendicular to the substrate from [532]_MnO_ to [431]_MnO_. In contrast, the greater *z*-axis shrinkage ratios observed in the BCC (20%) and pHCP (14%) superlattices were attributed to the softer NC building blocks, which allowed more flexible superstructures. GIWAXS also confirmed that the (011) plane of the Mn_3_O_4_ shell were parallel to the (111) plane of the MnO core in the nanooctahedra (fig. S22).

The experimental phase diagram of the 3D assembly of PS-grafted MnO@Mn_3_O_4_ nanooctahedra was constructed by systematically varying NC size and PS-PEHA molecular weight (fig. S23 and table S4). For 28-nm NCs, BCC superlattices formed with PS-PEHA ligands between 3.1 and 20.2 kDa, while pHCP superlattices formed with 44.7-kDa ligands. For 40-nm NCs, Minkowski structures were observed with ligands between 3.1 and 8.3 kDa, and BCC structures with ligands between 13.3 and 44.7 kDa. For 50-nm NCs, Minkowski structures formed with 3.1- to 13.3-kDa ligands, while BCC structures formed with 20.2- to 44.7-kDa ligands. These results indicate that the transition from Minkowski to BCC occurs with increasing ligand molecular weight or decreasing NC size. Previous studies support these trends: BCC structures are commonly observed in small nanooctahedra (e.g., <20 nm) grafted with short alkyl ligands (~2 nm), such as OA-capped Pt_3_Ni NCs (*43*) or PbS NCs (*41*). In contrast, Minkowski structures are often found in larger NCs, such as 300-nm Ag NCs with 30-kDa polyvinylpyrrolidone ligands (*7*, *40*) or 60-nm Au NCs with short cetylpyridinium chloride ligands (*38*).

**Fig. 4.**
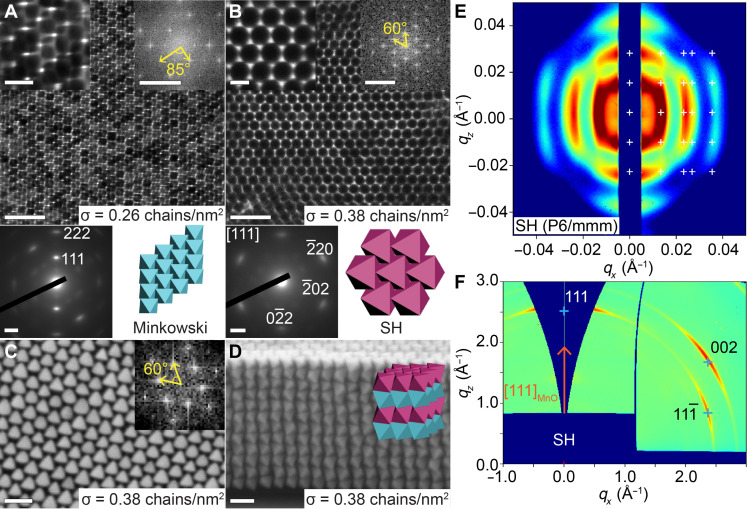
Phase transition in 3D superlattices of PS-grafted MnO@Mn_3_O_4_ nanooctahedra achieved by tuning ligand grafting density. (**A** and **B**) Characterization of the Minkowski (A) and SH (B) superlattices assembled from 50-nm MnO@Mn_3_O_4_ nanooctahedra functionalized with 8.3-kDa PS ligands at grafting densities of 0.26 chains/nm^2^ (A) and 0.38 chains/nm^2^ (B). Each panel shows a low-magnification TEM image (top), a high-magnification TEM image (top-left inset), the corresponding FFT pattern (top-right inset), the WAED pattern (bottom-left inset), and a structural model (bottom-right inset). Scale bars, 200 nm for low-magnification TEM images, 50 nm for high-magnification TEM images, 2 nm^−1^ for WAED patterns, and 0.05 nm^−1^ for FFT patterns. (**C** to **F**) Top-view SEM images (C) with the corresponding FFT pattern (inset), cross-sectional SEM image (D) with a structural model (inset), GTSAXS (E) and GIWAXS patterns (F) of the SH superlattice. Scale bars, 100 nm [(C) and (D)].

In addition to polymer ligand chain length, ligand grafting density is another key factor in governing the self-assembly phase behavior. For 8.3-kDa PS-grafted 50-nm nanooctahedra, increase in the grafting density from 0.26 to 0.38 chains/nm^2^ (fig. S24) induced a structural transition from the Minkowski phase to a SH phase (Fig. 4, A and B, and fig. S25). The FFT pattern of the SH superlattice exhibited hexagonal symmetry, while the corresponding WAED pattern showed six {220}_MnO_ diffraction spots belonging to the [111]_MnO_ zone axis. Correlation between the FFT and WAED patterns confirmed the orientational order of the nanooctahedra, with the (111)_MnO_ plane aligned parallel to the (001)_SL_ plane (fig. S25, D and F). Cross-sectional SEM imaging revealed that each nanooctahedron was in near-perfect face-to-face contact with two adjacent nanooctahedra across different (001)_SL_ layers, resulting in ABAB stacking of hexagonally close-packed (001)_SL_ plane (Fig. 4, C and D). Further structural analysis of the SH superlattice using GTSAXS/GIWAXS confirmed the translational and orientational order of the nanooctahedra (Fig. 4, E and F, and fig. S26). The SH structure was characterized by [001]_SL_ and [111]_MnO_ directions perpendicular to the substrate, with lattice constants *a* = *b* = 55.0 nm and *c* = 100.0 nm. The SH phase was previously identified as a minor phase coexisting with the Minkowski phase (*7*, *38*). However, this work demonstrates that by precisely controlling the PS-PEHA ligand grafting density of MnO@Mn_3_O_4_ nanooctahedra, phase-pure Minkowski and SH superlattices can be selectively obtained using the same NC core.

**Fig. 5.**
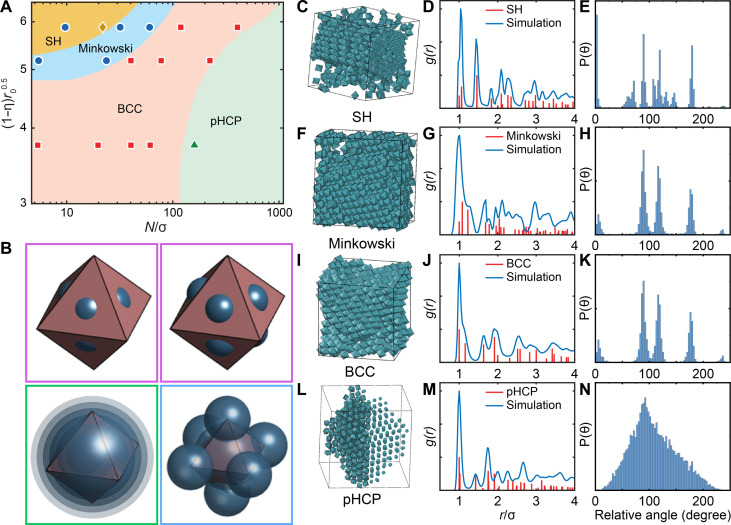
Phase diagram and structural analysis of 3D superlattices assembled from polymer-grafted MnO@Mn_3_O_4_ nanooctahedra. (**A**) Phase diagram of 3D superlattices as a function of key parameters, combining experimental data points with theoretically predicted equilibrium structures obtained using ALT, represented by the solid background colors. (**B**) CG computational model for PS-grafted nanooctahedra. The colored borders represent the corona morphologies defined in Fig. 1L. (**C** to **N**) Monte Carlo simulation results of the self-assembly of CG nanooctahedra into SH (C to E), Minkowski (F to H), BCC (I to K), and pHCP (L to N) superlattices. Left to right: (C), (F), (I), and (L) present simulation snapshots; (D), (G), (J), and (M) compare the radial distribution function g(r) between perfect and self-assembled lattices; and (E), (H), (K), and (N) show the distributions of relative orientational alignment between nanooctahedra within the self-assembled lattices. For the pHCP structure in (L), we omitted the rendering of the particle geometry and visualized each particle as a sphere for the right half of the box to better highlight spatial ordering between particles.

To provide a physical picture of the microscopic driving forces underlying the experimentally observed assemblies, we performed ALT calculations for 3D lattices. The theory-predicted phase diagram spanning the entire experimental phase space is shown in Fig. 5A, overlaid with experimental data and plotted using the same composite axes as the 2D phase diagram. Similar to 2D predictions, conformal coronas favored face-to-face aligned structures. However, in the 3D limit, there were two types of face-to-face aligned morphologies: SH and Minkowski lattices. In the Minkowski structure, all eight facets of each octahedron shared partial face-to-face contact with neighbors. In contrast, the SH structure featured more symmetric interactions, with two opposite facets forming 100% face-to-face contact along the *c* axis and the remaining six facets sharing partial contacts in the *ab* plane. ALT predictions indicated that the SH phase dominated at the shortest chain lengths, highest grafting densities, and largest NC edge lengths. Here, the grafted chains behaved like oligomeric ligands, creating “fuzzy” octahedra with strong face-to-face directional attraction arising from both polymer-mediated interactions and core-mediated directional entropic forces (*72*, *78*). Slightly longer chains softened the conformal corona morphology, weakening the synergistic interplay between core-mediated and polymer-mediated interactions. This reduction in the driving force favoring strong face-to-face alignments between NCs ultimately shifted the assembly toward the Minkowski lattice, which is characterized by partial face-to-face contacts. These results suggest that engineering strong directional interactions can effectively separate the coexistence of SH and Minkowski phases observed in prior studies. A discrepancy between theory and experiments was noted in the top-left region of the phase diagram (Fig. 5A), where experiments observed a Minkowski lattice, but theory predicted SH as the ground state. Inspection of ALT free-energy calculations revealed small energy differences between phases in this regime (fig. S27), suggesting that kinetic effects may favor the less orientation-restrictive Minkowski lattice.

As the corona transitioned to a convex morphology, the spherical cap–like corona motifs at the facets further weakened face-to-face interactions but did not entirely disrupt the preferential alignments inherent to the NC core geometry. In addition, longer polymeric grafts extended the range of polymer-mediated interactions. Consequently, the equilibrium assembly shifted to the BCC lattice, where each nanooctahedron leveraged long-ranged polymer interactions to stabilize vertex-to-vertex and edge-to-edge contacts with neighboring NCs. These interactions offsetted the increased energetic cost of compressing longer polymer chains in regions of the BCC lattice exhibiting face-to-face alignments, thereby stabilizing the entire assembly. In short, NCs with convex coronas leveraged long-range polymer interactions to counterbalance the energetic penalties of chain compression that ultimately produced a phase transition from the SH and Minkowski lattices to the BCC phase.

Transitioning to a concave corona morphology where chains preferentially partitioned to the NC vertices introduced opposing interaction motifs on each NC: core-mediated entropic forces favoring face-to-face alignment and corona-driven forces favoring vertex-to-vertex alignment. These opposing interactions canceled each other and drove polymer-grafted NCs to behave akin to isotropically interacting soft spheres. Such systems are well known in the literature to assemble into rotator HCP phases (*19*). Last, in regimes near the boundary between convex and concave morphologies (Fig. 1L), we observed the formation of the BCC structure in both experiments and theoretical predictions. This delayed transition in equilibrium assembly structure again reflected the limit where corona-mediated vertex interactions did not fully break the intrinsic preference for face-to-face alignment imposed by the NC core geometry. As a result, polymer-functionalized NCs preserved the BCC motif, where both vertex-to-vertex and face-to-face interactions coexisted within the self-assembled lattices. Analogous to the 2D case, we validated corona morphology transitions by measuring vertex-to-vertex distances between nanooctahedra in the BCC superlattice (fig. S28 and table S5). The significant increase in normalized vertex-to-vertex distances with polymer chain length indicates enhanced polymer density at particle vertices, creating additional steric repulsion that pushes particles further apart. This directly supports the predicted shift from conformal/convex to concave corona morphologies.

The strong agreement between theory and experiments for both 2D and 3D assemblies demonstrated that phase transitions between SH, Minkowski, BCC, and pHCP superlattices were driven by the microscopic modulation of polymer distribution around the octahedral NC cores. To test these insights, we developed a coarse-grained (CG) Monte Carlo model for PS-grafted MnO@Mn_3_O_4_ nanooctahedra, explicitly incorporating corona shapes predicted to drive assembly into each superlattice (Fig. 5B). The CG model used Lennard-Jones (LJ) interaction sites to capture the favored directional interactions defined by the corona shapes. Conformal coronas, favoring strong face-to-face interactions, were modeled as face-patchy octahedra. To simulate the transition from SH to Minkowski phases, patch sizes at each face were increased to soften patch-patch interactions. For the SH/Minkowski to BCC transition, corresponding to a shift from conformal to convex coronas, the model used spherically symmetric LJ interactions centered at each NC’s origin, with the LJ sphere diameter set to the insphere diameter of the octahedron, mimicking the soft, spherical convex coronas. To capture concave coronas, the model incorporated chain partitioning at edges and vertices by placing LJ beads at the six octahedral vertices and expanding the LJ sphere diameter from the insphere (used for convex coronas) to the midsphere of the octahedron. Simulation parameters and protocols are detailed in Materials and Methods. All MC simulations were performed using HOOMD-blue.

Simulation results demonstrated that our CG models of PS-grafted MnO@Mn_3_O_4_ nanooctahedra, parameterized using theoretical predictions of polymer distribution, successfully self-assembled into the experimentally observed superlattices corresponding to each corona morphology (Fig. 5, C, F, I, and L). Spatial ordering was confirmed by calculating the g(r) for each self-assembled lattice and comparing it with the g(r) of the perfect lattice (Fig. 5, D, G, J, and M). To capture the orientational ordering observed in experiments, we computed the relative orientations of CG octahedra with their neighbors (fig. S29). The angular distribution P(θ) showed high orientational order in the SH, Minkowski, and BCC phases and rotational disorder in the HCP phase, matching experimental results (Fig. 5, E, H, K, and N). Visualization of the self-assembled structures further corroborated these spatial and orientational orderings (movies S2 to S5). These findings confirmed that the experimentally observed phase behaviors were driven by variations in polymer distribution on the NC surface. Furthermore, the equations used to predict corona morphology readily generalize to diverse NC shapes. Together, these findings highlight that our theoretical framework for corona morphology and lattice prediction is a robust tool for a priori design of polymer-grafted NCs based on experimentally relevant parameters.

## DISCUSSION

In summary, we studied phase transitions in polymer-grafted nanooctahedron superlattices by systematically varying NC edge length, polymer ligand length, and ligand grafting density. In 2D superlattices, translational ordering was preserved, while orientational ordering declined with increasing polymer chain length or decreasing nanooctahedron edge length. In 3D superlattices, transitions from Minkowski to BCC and pHCP phases were driven by increasing polymer ligand length, with thresholds determined by the nanooctahedron edge length, while transitions from Minkowski to SH structures were achieved by increasing ligand grafting density. To understand the driving forces behind these transitions, we predicted polymer corona shapes on the basis of experimental parameters and traced how changes in corona morphology influenced phase behaviors. These predictions closely aligned with experimental results, validating the robustness of our approach. Our work establishes a direct link between microscopic phenomena, such as polymer conformation and corona morphology, and macroscopic orientational and spatial ordering in superlattices. Recognizing corona morphology as a primary driver of NC assembly can guide CG modeling, facilitating studies of assembly kinetics and enabling targeted design of out-of-equilibrium or metastable morphologies. Last, our findings highlight macromolecular ligand engineering as a powerful strategy for tuning the interplay between shape- and polymer-driven interactions, particularly in the self-assembly of anisotropic NCs. This work demonstrates the potential of polymer-grafted anisotropic NCs as versatile building blocks, capable of leveraging entropy and enthalpy to sculpt hierarchical superstructures and next-generation metamaterials with tunable collective properties.

## MATERIALS AND METHODS

### Chemicals

Styrene (≥99%), ethyl α-bromoisobutyrate (98%), copper(II) bromide (99%), Tris[2-(dimethylamino)ethyl]amine (97%), tin(II) 2-ethylhexanoate (92.5 to 100%), PEHA (technical grade), aluminum oxide (activated, neutral, Brockmann I), triethylamine (≥99.5%), OA (technical grade, 90%), DEG (ReagentPlus, ≥99.0%), and *N*,*N*-dimethylformamide (DMF; anhydrous, 99.8%) were purchased from Sigma-Aldrich. Calcium hydride (CaH_2_; 90 to 95%) and manganese(II) acetate (MnAc_2_; anhydrous, 98%+) were purchased from Alfa Aesar. Tri-*n*-octylamine (TOA; >97.0%) was purchased from TCI America. Unless otherwise noted, all chemicals were used as received. Styrene was purified by extraction with 10 wt % sodium hydroxide solution to remove inhibitors, followed by distillation over CaH_2_ at 35°C under reduced pressure.

### Synthesis of MnO@Mn_3_O_4_ core-shell nanooctahedra

MnO nanooctahedra were synthesized following a previously reported method with modifications (*58*). For MnO nanooctahedra with an edge length of 28 nm, 8 mmol of MnAc_2_, 6.6 ml of OA, and 30 ml of TOA were combined in a 100-ml three-neck flask at room temperature. The mixture was heated under vacuum at 120°C for 2 hours with stirring, forming a clear, pale-yellow solution. After purging the system with N_2_, the reaction mixture was rapidly heated to 324°C under a continuous N_2_ flow. Once the solution color changed from pale yellow to green, the reaction was held at this temperature for another 30 min.

MnO nanooctahedra with edge lengths of 40 and 50 nm were synthesized at reaction temperatures of 314°and 305°C, respectively. After cooling the reaction mixture to room temperature, the MnO nanooctahedra were precipitated by adding isopropanol, followed by centrifugation at 4500 rpm for 3 min. The resulting precipitate was redispersed in 20 ml of chloroform, and another 20 ml of isopropanol was added before repeating the centrifugation step. The final product was redispersed and stored in 20 ml of chloroform under ambient conditions. Overnight, the color of the stock solution transitioned from green to brown, indicating the surface oxidation of MnO nanooctahedra into MnO@Mn_3_O_4_ core-shell nanooctahedra.

### Grafting PS-PEHA onto MnO@Mn_3_O_4_ core-shell nanooctahedra

A two-step ligand-exchange process developed by our group was used, which involves the removal of native OA ligands followed by grafting with PS-PEHA (*16*). The detailed synthesis of PS-PEHA is described in the Supplementary Materials.

In the first step, 1 ml of a 0.25 M acetonitrile solution of Et_3_OBF_4_ was injected into 2 ml of hexane solution containing MnO@Mn_3_O_4_ nanooctahedra (7 mg/ml). The mixture was stirred at room temperature for 1 hour to facilitate the transfer of NCs from the upper hexane phase to the lower acetonitrile phase. After removing the upper hexane layer, the ligand-stripped nanooctahedra in acetonitrile were precipitated by adding 2 ml of toluene, followed by centrifugation at 4500 rpm for 3 min. The resulting precipitate was redispersed in 1 ml of DMF, then 3 ml of toluene was added, and the mixture was centrifuged again at 4500 rpm for 3 min. Last, the purified, ligand-stripped nanooctahedra were redispersed in 3 ml of DMF, yielding a stock solution with a concentration of 3.3 mg/ml. All steps were conducted inside an N_2_-purged glovebox using anhydrous solvents.

In the second step, a measured amount of PS-PEHA was dissolved in 1.4 ml of tetrahydrofuran (THF). Then, 0.6 ml of NC solution containing 2 mg of ligand-stripped MnO@Mn_3_O_4_ nanooctahedra was injected into the PS-PEHA solution. After brief sonication for 2 s, the mixture was allowed to sit undisturbed for 24 hours. The PS-grafted NCs were precipitated using heptane, followed by centrifugation at 4500 rpm for 3 min. The resulting pellet was redispersed in 2 ml of THF, and the purification process was repeated once more. Last, the purified NCs were dispersed in 0.2 ml of toluene.

The feeding grafting density ($\sigma_{f}$) is defined as the ratio of the number of PS-PEHA ligands used during the ligand-exchange process to the total surface area of the MnO@Mn_3_O_4_ nanooctahedra, expressed as

$$\sigma_{f}=\frac{\sqrt{6}\times a\times \rho \times N_{A}\times m_{\text{PS}}}{18\times M_{n}\times m_{\text{NC}}}$$

where a is the edge length of the nanooctahedra, ρ is the bulk density of MnO (5.43 g/cm^3^) (*79*), $N_{A}$ is Avogadro’s constant, $M_{n}$ is the number-averaged molecular weight of PS-PEHA, $m_{\text{PS}}$ is the mass of PS-PEHA ligands used for ligand exchange, and $m_{\text{NC}}$ is the mass of the inorganic cores of the MnO@Mn_3_O_4_ nanooctahedra. Given that the amount of residual OA ligands is negligibly small in the ligand-stripped nanooctahedra, the actual grafting density of PS-PEHA (σ) can be calculated using the following equation

$$\sigma =\frac{\sqrt{6}\times a\times \rho \times N_{A}\times X}{18\times M_{n}\times \left(1-X\right)}$$

where X is the mass fraction of PS-PEHA ligands as determined by TGA, and all other parameters are the same as those defined in the previous equation. To account for the mass increase due to surface oxidation of rock-salt MnO into spinel Mn_3_O_4_ during the TGA measurement, X is calculated as

$$X=1-\frac{m_{\text{PS}-\text{capped}}}{m_{{\text{BF}}_{4}^{-}-\text{capped}}}$$

Here, $m_{\text{PS}-\text{capped}}$ represents the final weight percentage of PS-grafted MnO@Mn_3_O_4_ nanooctahedra after the TGA run, and $m_{{\text{BF}}_{4}^{-}-\text{capped}}$ (>100%) is the final weight percentage of ${\text{BF}}_{4}^{-}$-capped MnO@Mn_3_O_4_ nanooctahedra after the TGA run. The values of $\sigma_{f}$ and σ values for different samples are summarized in table S2.

### Self-assembly of PS-grafted MnO@Mn_3_O_4_ nanooctahedra into superlattices

PS-grafted nanooctahedron superlattices were prepared using a liquid-air interfacial assembly method (*18*, *70*). To prepare 2D superlattices, 20 μl of toluene solution containing PS-grafted MnO@Mn_3_O_4_ nanooctahedra (10 mg/ml) was carefully drop-cast onto the surface of DEG in a Teflon well (1.5 cm by 1.5 cm by 1.5 cm). The Teflon well was then promptly covered with two glass slides to slow down solvent evaporation. For the preparation of 3D superlattices, the same procedure was followed, but 200 μl of the toluene solution containing PS-grafted MnO@Mn_3_O_4_ nanooctahedra was used. After allowing the solvent to evaporate completely (12 hours for 2D monolayers and 48 hours for 3D superlattices), the assembled NC films were carefully transferred onto carbon-coated Cu TEM grids or silicon wafers. The transferred films were then dried in a vacuum oven to eliminate any residual DEG.

### Characterization of materials

Low-magnification TEM images and WAED were acquired using a JEOL JEM 1400Plus microscope equipped with a LaB_6_ filament, operating at 120 kV. HAADF-STEM images and NBED and EELS spectra were obtained with a Cs-corrected JEOL NeoARM TEM equipped with a cold field emission gun, operating at 200 kV. The tilt series for TEM tomography reconstruction were acquired on a JEOL JEM-2100Plus TEM, operating at 200 keV. The tilting angles range from −51° to 53° with a step of 2°. The alignment of the tilt series was performed by the Composer software using cross-correlation. 3D tomography reconstruction of the tilt series was carried out with software using simultaneous iterative reconstruction technique algorithm with 20 iterations. Intensity thresholding and volume rendering were done using the Visualizer-evo software.

SEM images were captured using a Carl Zeiss Auriga 60 FIB-SEM, operating at 3 kV. To record cross-section SEM images, superlattice film–deposited Si wafers were cracked into two halves evenly by a wafer cutter, and the sample stage was tilted by 54° during the image acquisition process. Powder x-ray diffraction (XRD) patterns were recorded using a PANalytical Empyrean XRD, operating at 45 kV and 40 mA. XRD samples were prepared by drop-casting the NC solution onto single-crystalline silicon substrates. GTSAXS and GIWAXS measurements were conducted at the Complex Materials Scattering (CMS) beamline (11-BM) of the National Synchrotron Light Source (NSLS) II, Brookhaven National Laboratory. The x-ray beam was monochromated to 13.5 keV and had a beam size of 0.2 mm by 0.05 mm. The incident angle for GTSAXS/GIWAXS was set to 0.5 degrees. Two detectors, Pilatus 2M and Pilatus 800K, were positioned at 5 m and 259 mm from the sample, respectively, to simultaneously collect GTSAXS and GIWAXS data. Data reduction was performed using the SciAnalysis software, and the 2D scattering patterns were indexed with the custom-developed indexGIXS program.

FTIR spectra were acquired in transmission mode using a Bruker VERTEX 70v FTIR spectrometer at a spectral resolution of 4 cm^−1^. FTIR transmittance for different samples was normalized to the mass per unit area of the NC inorganic cores in the deposited film, assuming a uniform film thickness. TGA was conducted using a TA Instruments Q5000 system. Approximately 2 mg of NCs were loaded into a platinum pan, which was first heated to 120°C for 30 min to remove residual solvents. The temperature was then increased to 700°C at a constant rate of 10°C/min under a nitrogen atmosphere. Data analysis was performed using the TA Universal Analysis software. GPC data were obtained using an Agilent GPC50 system equipped with a refractive index detector and three columns in series [Agilent PLgel 5-μm Mixed-C, 10-μm Mixed-B (300 mm by 7.5 mm), and PLgel 5-μm Guard (50 mm by 7.5 mm)]. THF was used as the eluent at a flow rate of 1 ml/min. The instrument was calibrated with commercial PS standards.

### Monte Carlo simulations of NC assembly

The CG parameterization for PS-grafted nanooctahedra is described below. All interactions between spherical patches, which represent CG polymer-polymer interactions, are governed by the LJ potential given by

V(r)=4ε[(σr)12−(σr)6]

For the conformal corona that forms the SH lattice, the CG parameters are ε=6 and $\sigma =0.37\sigma_{\text{in}}$, where $\sigma_{\text{in}}$ is the diameter of the insphere of the octahedral core. For the conformal corona that forms the Minkowski lattice, the CG parameters are ε=5 and $\sigma =0.30\sigma_{\text{in}}$. For conformal coronas, spherical patches are positioned at the midpoints of each octahedral face. The patch origins are located at distances of $0.55\sigma_{\text{in}}$ and $0.80\sigma_{\text{in}}$ from the center of the octahedral core for the SH and Minkowski lattices, respectively.

For the convex corona, a single CG LJ particle is placed at the center of the octahedron, with parameters ε=10 and $\sigma =\sigma_{\text{in}}$. This configuration generates a spherically symmetric attractive field around the core, effectively capturing the soft, convex corona morphology.

For the concave corona, seven spherical LJ particles are used. Six LJ particles are placed at the vertices of the octahedron, each with $\sigma =0.5\sigma_{\text{in}}$. The seventh LJ particle is placed at the center of the octahedron, analogous to the convex corona, but with $\sigma =\sigma_{\text{mid}}$, where $\sigma_{\text{mid}}$ represents the midsphere diameter of the octahedron. All spherical LJ particles are assigned an interaction strength of ε=1.5.

Simulations are initialized with particles randomly distributed in a large simulation box at a volume fraction below 0.05. The system is then compressed to a volume fraction of 0.35 and equilibrated over 107 Monte Carlo moves. To facilitate rapid equilibration, we tuned the acceptance ratios for both translational and rotational moves to 0.3. LJ interactions between all CG spherical patches are incorporated using the Metropolis criterion. To overcome the energetic barriers associated with nucleation and growth, the SH and Minkowski lattices are seeded with a small 32-particle crystal of their respective structures. All simulations are conducted using the HOOMD-blue simulation engine, and structural and orientational characterizations are performed with the freud analysis package.

### Theory prediction of PS-grafted nanoactrahedra self-assembly

To connect corona morphology with the macroscopic assembly behaviors observed in NCs, we use ALT (*20*, *65*, *71*). Briefly, ALT defines a noninteracting reference state of “hard particles,” represented by the predicted corona morphology, which occupy the lattice sites of a given crystal structure. We then computed the potential of mean force (PMF) between two PS-grafted NCs as a function of their center-to-center distance for a given relative orientation, accounting for core-core, core-graft, and graft-graft interactions. The computed PMF serves as an effective interaction potential, V(r), between NCs in the lattice. Using ALT, we calculated the excess free energy of lattice formation using ΔG=−kTlnK, where $K=\prod_{i}\sum_{j}\frac{\rho^{s_{\text{ij}}}}{s_{\text{ij}}!}\int f_{\text{ij}}^{s_{\text{ij}}}\left(r\right)g_{\text{ij},c}\left(r\right)d\overset{⃑}{r}$, $f\left(r\right)=e^{-\beta V\left(r\right)}-1$ is the Mayer-f function, ρ is the system density, $g_{c}\left(r\right)$ is the radial distribution function for the a given crystalline lattice, s is the coordination number of the ideal lattice, and i and j denote the types of interactions and particles in the system. To construct the phase diagram, we computed g(r) for each lattice (Minkowski, BCC, pHCP, and SH) and determined the set of particle orientations that yielded the lowest ΔG. This is then identified as the equilibrium configuration for a given lattice, with its corresponding ∆G being the ground state excess free energy of formation. Selecting the lattice with the lowest ∆G for each point in phase space produced the equilibrium phase diagrams reported in Figs. 2K and 5A.

## Supplementary Materials

### The PDF file includes:

Supplementary Text Figs. S1 to S29 Tables S1 to S5 Legends for movies S1 to S5 References
