# Supplementary material for: Superstructural phase transitions in polymer-grafted nanooctahedra
Source: Sci Adv. 2025 Jul 18;11(29):eadw2740. doi: 10.1126/sciadv.adw2740 (PMC12273772; doi:10.1126/sciadv.adw2740)
Supplement: Supplementary file 1 — Supplementary Text Figs. S1 to S29 Tables S1 to S5 Legends for movies S1 to S5 References [file sciadv.adw2740_sm.pdf]

Science Advances
AAAS

Supplementary Materials for

Superstructural phase transitions in polymer-grafted nanooctahedra

Baixu Zhu *et al.*

Corresponding author: Ruipeng Li, rli@bnl.gov; Thi Vo, tvo12@jhu.edu; Xingchen Ye, xingye@indiana.edu

Sci. Adv. **11**, eadw2740 (2025)
DOI: 10.1126/sciadv.adw2740

The PDF file includes:

Supplementary Text
Figs. S1 to S29
Tables S1 to S5
Legends for movies S1 to S5
References

Other Supplementary Material for this manuscript includes the following:

Movies S1 to S5

Supplementary Text

Data analysis of electron energy loss spectroscopy spectra.

1. Determination of manganese valence states

The electron energy loss spectroscopy (EELS) spectra in this study were collected with a dispersion of 0.1 eV/channel. Core and shell EELS spectra were extracted from the EELS spectrum image using the Mn L_3 and L_2 white lines, which correspond to the electronic transitions $2p_{3/2}\rightarrow 3d_{3/2,5/2}$ and $2p_{1/2}\rightarrow 3d_{3/2}$, respectively. The ratio of the integrated intensities of L_3 to L_2, $I(L_3)/I(L_2)$, was calculated using the "double arctan EELS background" script in Gatan Digital Micrograph software. (*80*) The $I(L_3)/I(L_2)$ ratio was approximately 3.93 for the core and 2.91 for the shell. Comparison of these values with literature reports[4] confirmed that the core and shell valence states in the nanocrystals correspond to MnO and Mn_3O_4, respectively. This conclusion is consistent with the results from STEM imaging and corresponding nanobeam electron diffraction (NBED) analysis.

2. Analysis of the distribution of manganese valence state within MnO@Mn_3O_4 nanooctahedra

The distribution of manganese valence states within a MnO@Mn_3O_4 nanocrystal was determined from EELS spectrum imaging. Reference spectra for the white lines corresponding to MnO, Mn_3O_4, and the background were used as standards. Multiple linear least squares (MLLS) fitting was applied to the EELS spectrum image, enabling the spatial decomposition of the spectral signals. This analysis provided a detailed map of the manganese valence state distribution, clearly distinguishing the Mn^{2+}-dominated MnO core from the Mn^{3+}/ Mn^{2+} mixed-valence Mn_3O_4 shell.

Synthesis of pentaethylenehexamine-terminated polystyrene

Pentaethylenehexamine-terminated polystyrene (PS-PEHA) was synthesized using a two-step method previously reported by our group (fig. S1). (*16*, *17*) In the first step, bromine-terminated polystyrene (PS-Br) was prepared through activators regenerated by electron transfer atom transfer radical polymerization (ARGET ATRP). In the second step, the bromine terminal group of PS-Br was substituted with PEHA, a multidentate nitrogen-containing ligand.

1. *Synthesis of PS-Br*.

In a typical synthesis of PS-Br with a molecular weight exceeding 8 kDa, styrene (100-600 eq, 43.6 mmol), ethyl α-bromoisobutyrate (EBIB) (1.0 eq), copper(II) bromide ($CuBr_2$) (0.01 eq) and tris[2-(dimethylamino)ethyl]amine (Me_6TREN) (0.1 eq) were added to a round-bottom flask. The mixture was purged with N_2 for 45 minutes, after which tin(II) 2-ethylhexanoate ($Sn(EH)_2$) was rapidly injected. The reaction was conducted at 90 °C with constant stirring for 17–22 hours. The polymerization was terminated by exposing the mixture to air while cooling the flask in an ice bath. For PS-Br with a molecular weight below 5 kDa, a similar procedure was followed. Styrene (30 eq, 21.8 mmol), EBIB (1.0 eq), $CuBr_2$ (0.01 eq), anhydrous toluene (25 mL) and Me_6TREN (0.1 eq) were combined in a round-bottom flask. After purging with N_2 for 45 minutes, $Sn(EH)_2$ was injected, and the reaction was carried out at 90 °C with stirring for 17–25 hours. The reaction was terminated by cooling the flask in an ice bath. Detailed experimental parameters are provided in table S1.

To purify the resulting polymers, the crude reaction mixture was diluted with tetrahydrofuran (THF), and the polymers were precipitated using methanol. The precipitate was collected via vacuum filtration and redissolved in THF. To remove residual copper-based catalysts, neutral

Al_2O_3 was added to the polymer-THF solution, and the mixture was stirred overnight. The neutral Al_2O_3 was subsequently removed by vacuum filtration, and the purified polymer was reprecipitated with methanol. The final precipitate was dried in a vacuum oven at 40 °C overnight.

2. *Conversion of PS-Br to PS-PEHA.*

In a typical reaction, PS-Br (1 eq), PEHA (50 eq), and triethylamine (100 eq) were dissolved in DMF and stirred at room temperature for 72 hours. The resulting PS-PEHA was precipitated using methanol and collected via vacuum filtration. The polymers underwent two additional purification cycles involving dissolution in THF followed by precipitation with methanol. The final product was dried in a vacuum oven at 40 °C overnight.

<u>Discussion on Fourier-transform infrared peak assignments for different nanocrystal samples.</u>

Fourier-transform infrared (FTIR) spectroscopy was employed to investigate the ligand composition of as-synthesized, BF_4^--capped, and PS-grafted MnO@Mn_3O_4 nanooctahedra. The analysis focused on three key spectral regions, each corresponding to specific functional groups and bonding characteristics:

1. *Region 1 (3200–2800 cm^{-1})*: This region is dominated by C-H stretching vibrations from alkyl chains. (*16*) Peaks in the range of 3200–3020 cm^{-1} (#1) are attributed to aromatic C-H stretching vibrations, which serve as characteristic signals for PS-grafted MnO@Mn_3O_4 nanooctahedra. In contrast, peaks in the range of 3000–2800 cm^{-1} (#2) correspond to saturated aliphatic C-H stretching vibrations, which are present in both as-synthesized OA-capped and PS-grafted MnO@Mn_3O_4 nanooctahedra. In BF_4^--capped samples, these peaks are significantly diminished, indicating the effective removal of OA ligands during ligand stripping.

2. *Region 2 (1600–1400 cm^{-1}) and Region 3 (1100–1000 cm^{-1})*: Peak assignments are described below.
   - 1600 cm^{-1} (#3): N-H bending vibrations from PEHA or C=C stretching vibrations from the aromatic rings in PS-PEHA. (*16*)
   - 1583 cm^{-1} (#4), 1492 cm^{-1} (#5) and 1452 cm^{-1} (#6): C=C stretching vibrations from the aromatic rings in PS-PEHA. Additionally, the peak at 1452 cm^{-1} can be attributed to CH_2 in-plane scissoring vibrations. (*16*)
   - 1558 cm^{-1} (#9): asymmetric stretching vibration of the bridging bidentate (O-C-O) group. (*81*)
   - 1436 cm^{-1} (#10): symmetric stretching vibration of the O-C-O group. (*16*)
   - 1066 cm^{-1} (#7) and 1028 cm^{-1} (#8): in-plane C-H bending vibrations of the aromatic rings in PS-PEHA. (*16*)

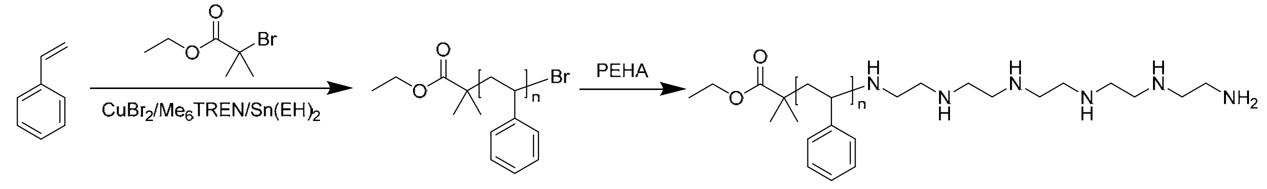

Fig. S1.

Synthesis scheme of PS-PEHA.

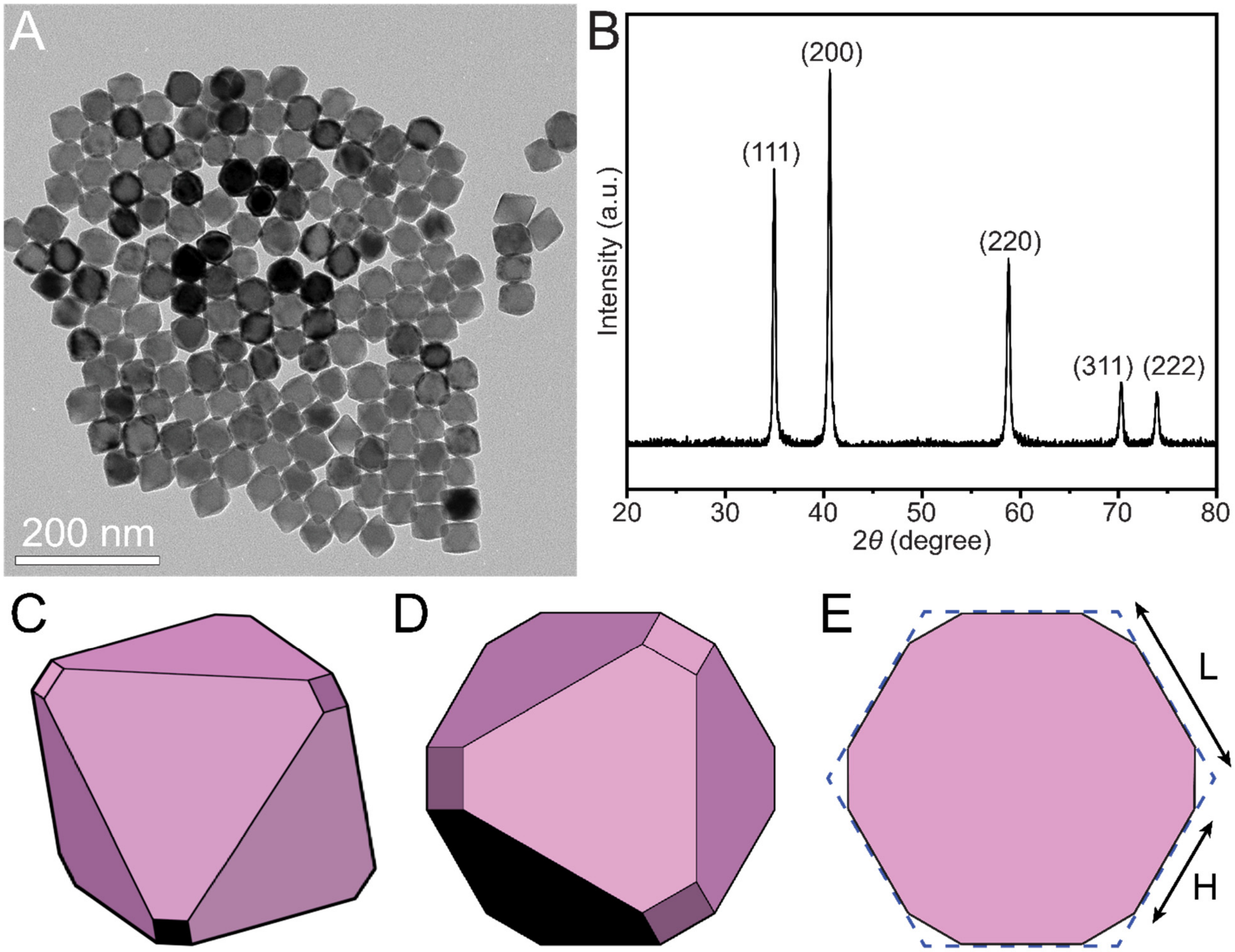

Fig. S2.

Structural characterizations of MnO@Mn_3O_4 nanooctahedra. (A, B) TEM image (A) and powder XRD pattern (B) of MnO@Mn_3O_4 nanooctahedra. (C, D) Schematics of a truncated MnO@Mn_3O_4 nanooctahedron depicted from various perspectives. (E) A projection view aligned along the [111] zone axis of the MnO core. The degree of truncation (η) is defined as $\eta = 1 - H/L$, where H represents the measured edge length, and L denotes the extrapolated edge length of the hexagon.

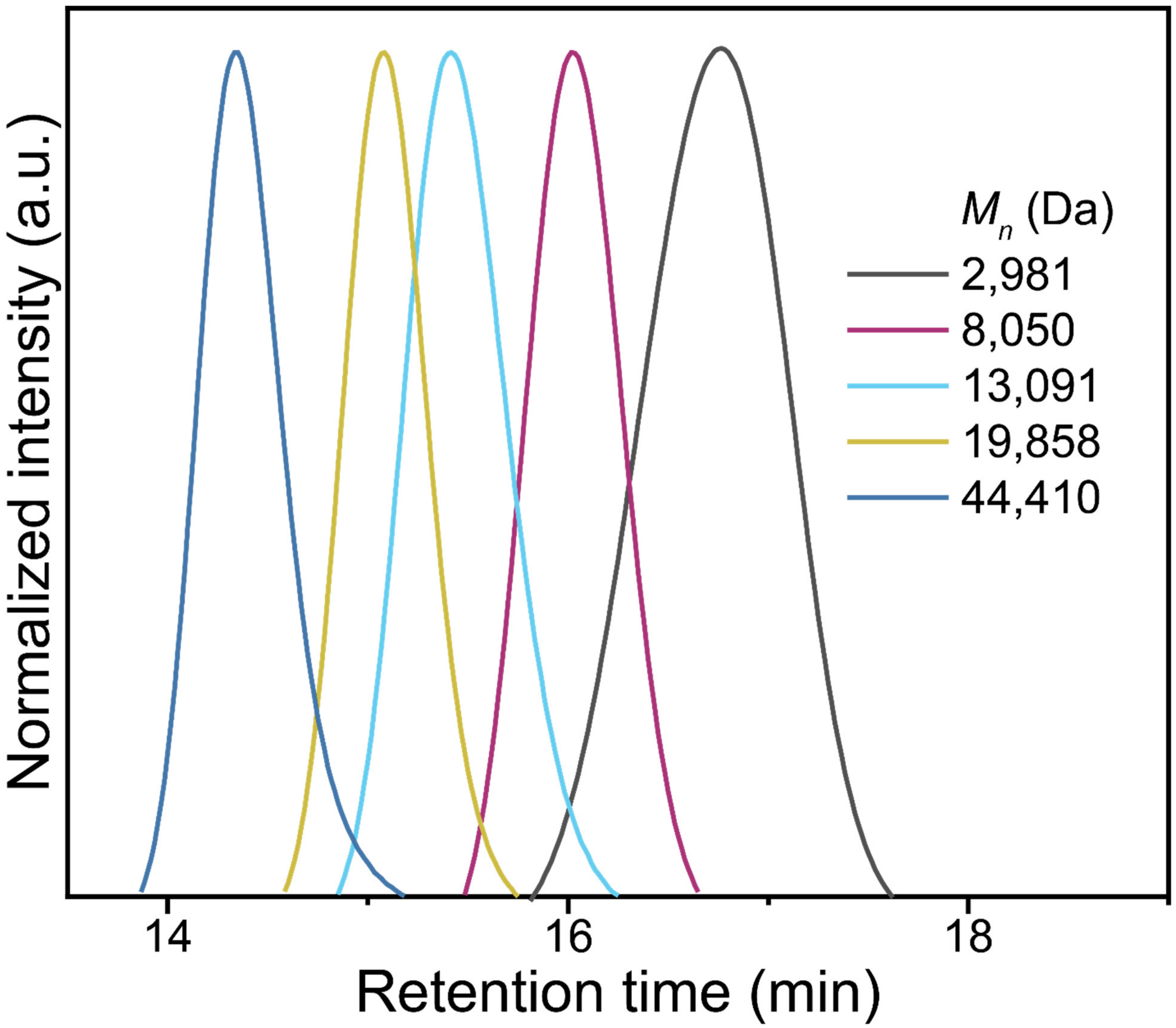

Fig. S3.
Normalized gel permeation chromatography traces of various PS-Br samples.

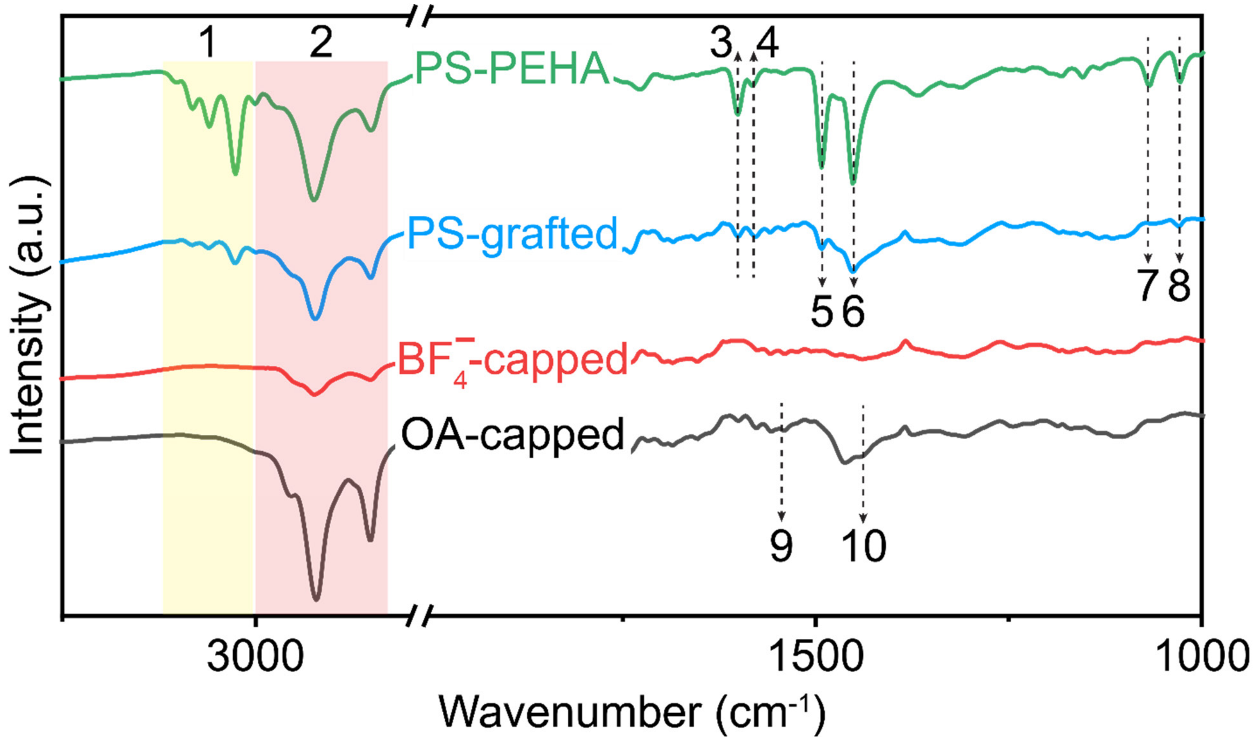

Fig. S4.

Representative FTIR spectra of as-synthesized, BF_4^--capped, and PS-grafted MnO@Mn_3O_4 nanooctahedra and PS-PEHA.

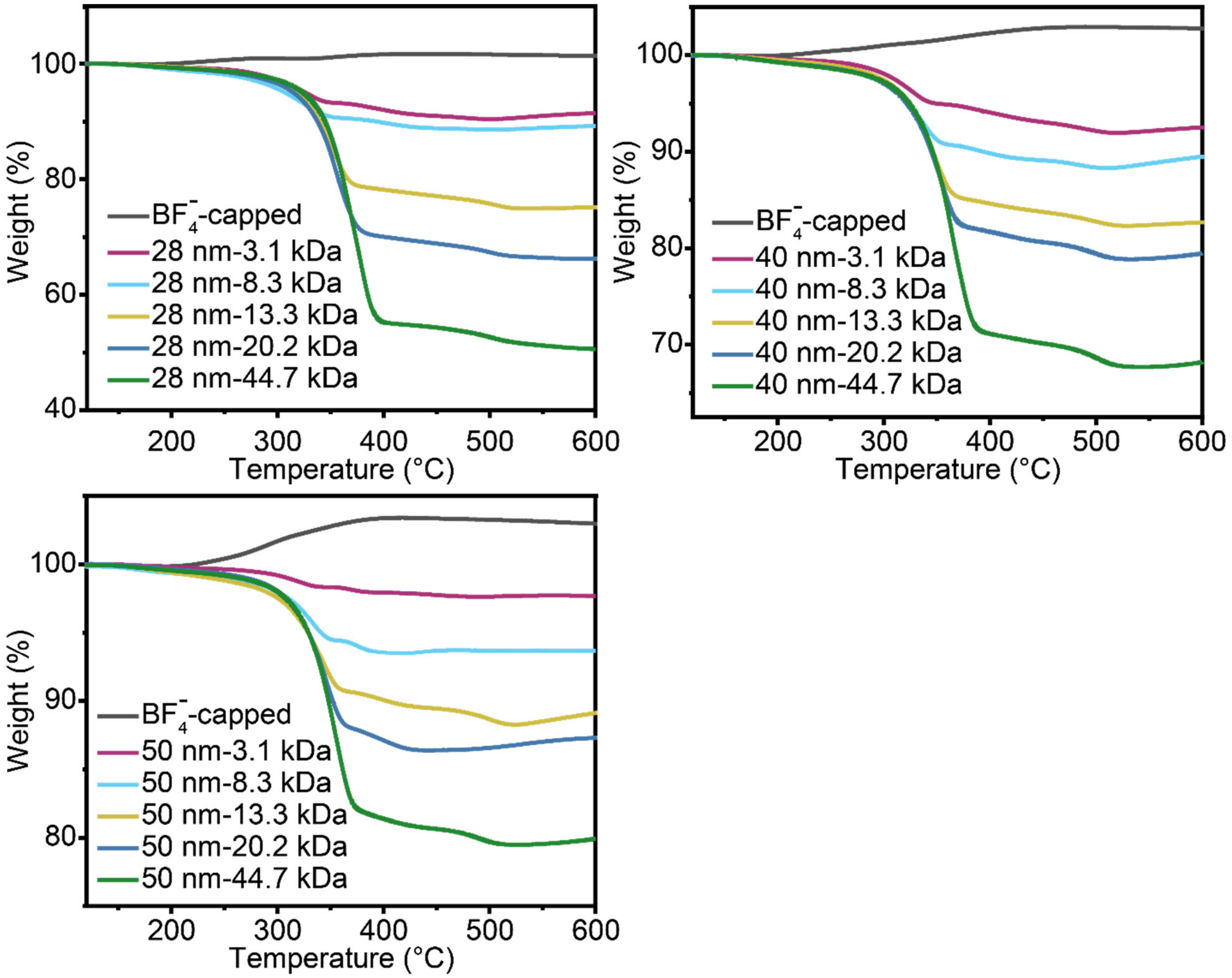

Fig. S5.

Thermogravimetric analysis (TGA) results of BF_4^--capped and PS-grafted MnO@Mn_3O_4 nanooctahedra. The edge lengths of the nanooctahedra and the number-average molecular weight (M_n) of the PS-PEHA ligands were indicated on the plots.

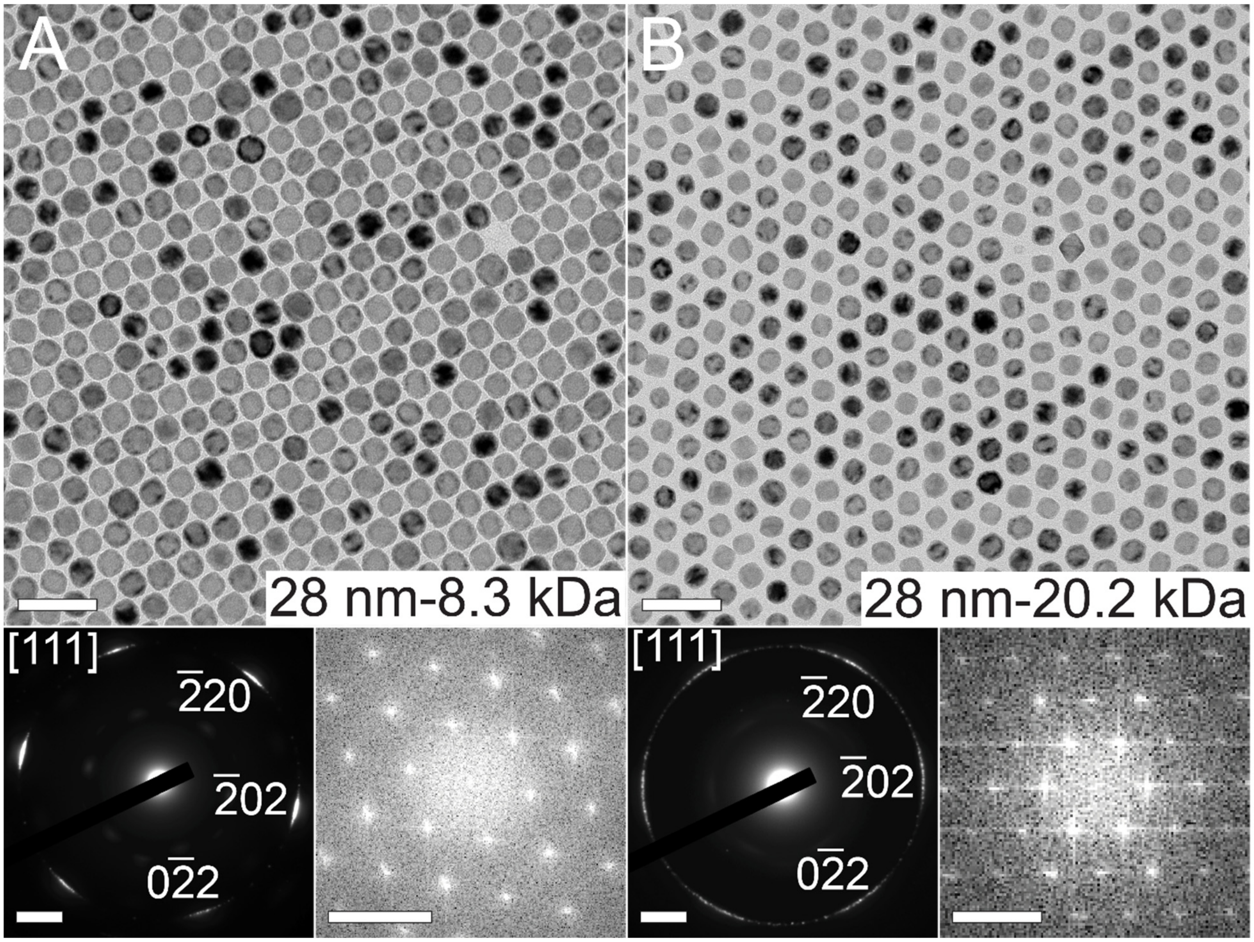

Fig. S6.

Superstructural phase transitions in 2D superlattices assembled from PS-grafted 28 nm $MnO@Mn_3O_4$ nanooctahedra. (Top) TEM images of 2D superlattices formed by 28 nm nanooctahedra functionalized with (A) 8.3 kDa and (B) 20.2 kDa PS-PEHA ligands. (Bottom left) Corresponding WAED patterns, and (bottom right) fast Fourier transform (FFT) patterns. Scale bars: 100 nm for TEM images, 2 nm^{-1} for WAED patterns, and 0.05 nm^{-1} for FFT patterns.

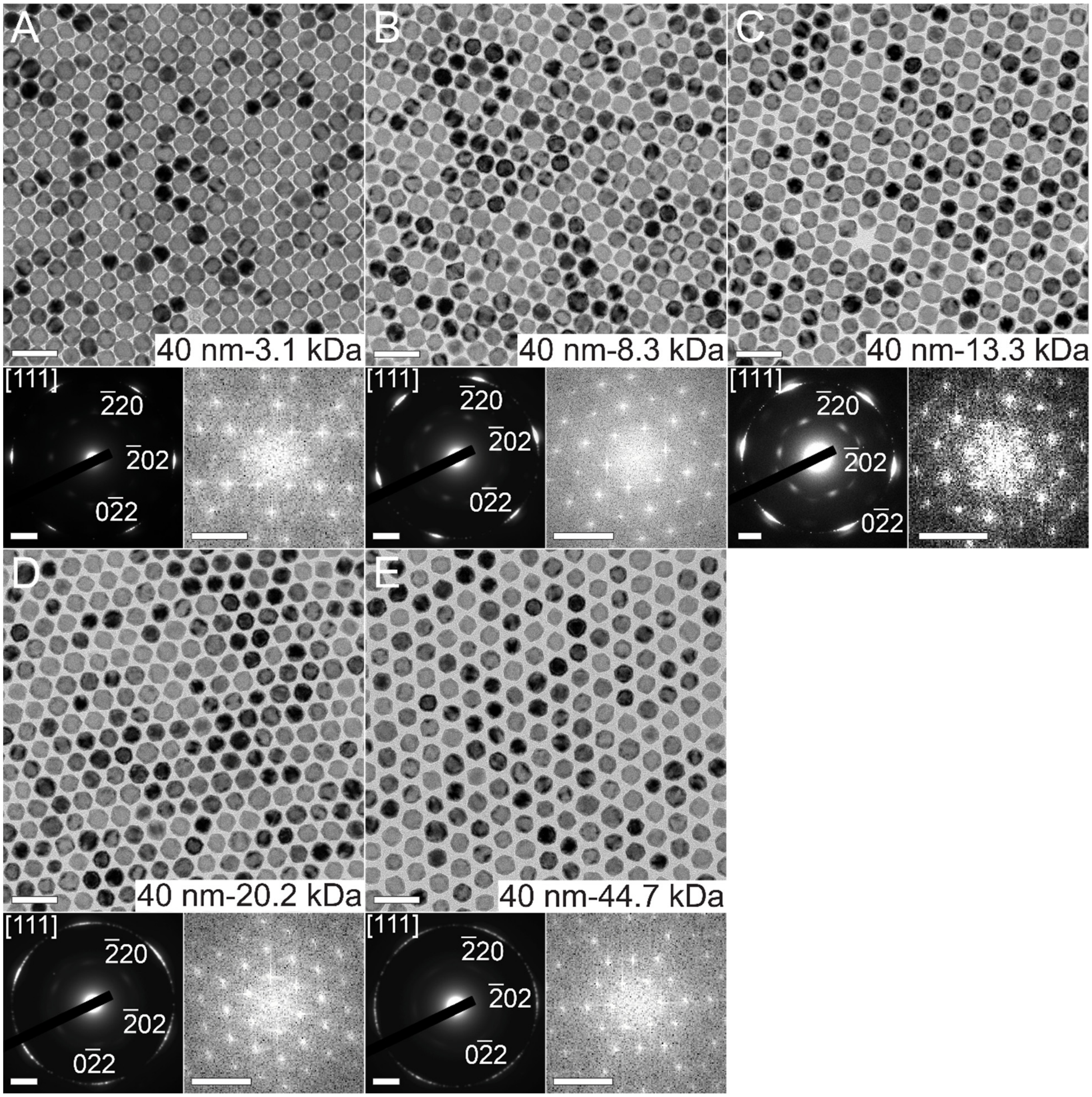

Fig. S7.

Superstructural phase transitions in 2D superlattices assembled from PS-grafted 40 nm $MnO@Mn_3O_4$ nanooctahedra. (Top) TEM images of 2D superlattices formed by 40 nm nanooctahedra functionalized with (A) 3.1 kDa, (B) 8.3 kDa, (C) 13.3 kDa, (D) 20.2 kDa, and (E) 44.7 kDa PS-PEHA ligands. (Bottom left) Corresponding WAED patterns, and (bottom right) FFT patterns. Scale bars: 100 nm for TEM images, 2 nm^{-1} for WAED patterns, and 0.05 nm^{-1} for FFT patterns.

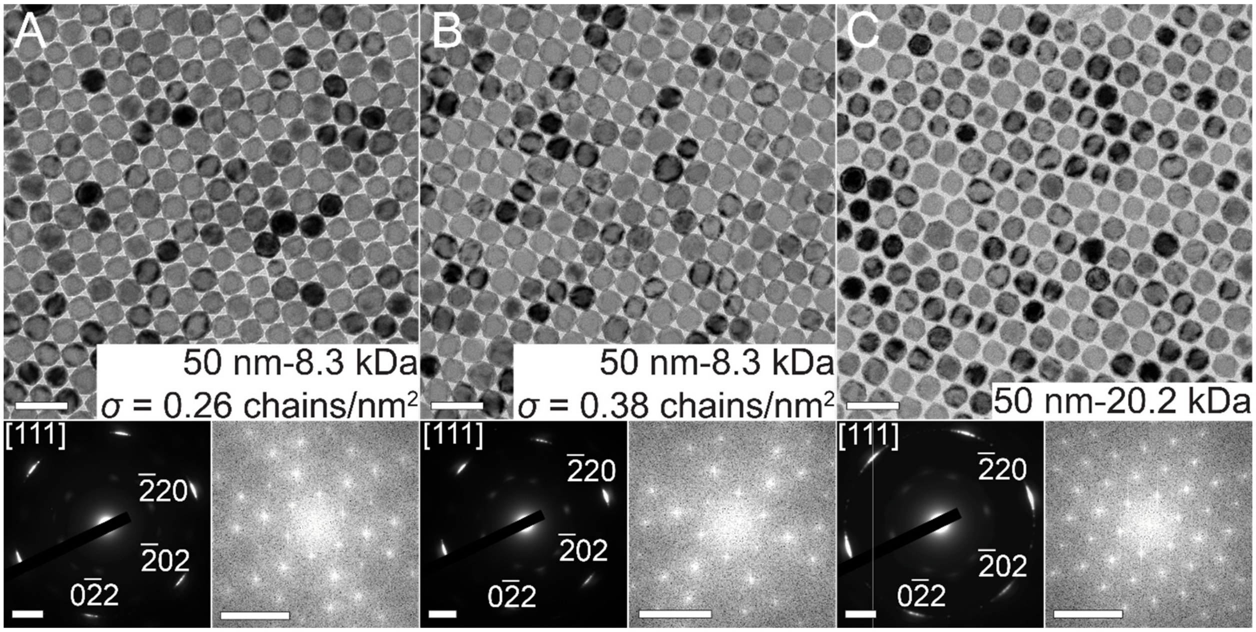

Fig. S8.

Superstructural phase transitions in 2D superlattices assembled from PS-grafted 50 nm MnO@Mn_3O_4 nanooctahedra. (Top) TEM images of 2D superlattices formed by 50 nm nanooctahedra functionalized with (A, B) 8.3 kDa PS-PEHA ligands at grafting densities of σ = 0.26 chains/nm^2 (A) and σ = 0.38 chains/nm^2 (B), and with 20.2 kDa PS-PEHA ligands (C). (Bottom left) Corresponding WAED patterns, and (bottom right) FFT patterns. Scale bars: 100 nm for TEM images, 2 nm^{-1} for WAED patterns, and 0.05 nm^{-1} for FFT patterns.

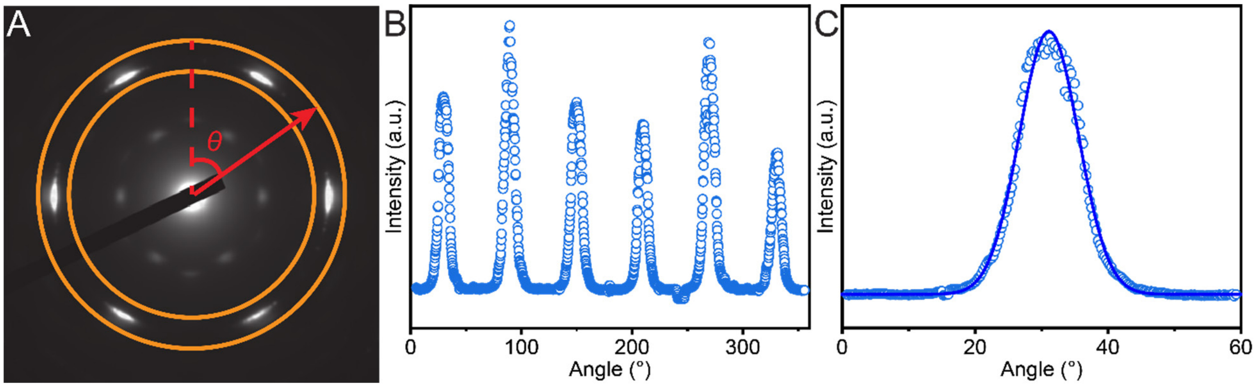

Fig. S9.

Extracting radial intensity profiles from WAED patterns. (A) Representative WAED pattern of a 2D superlattices of MnO@Mn_3O_4 nanooctahedra with orientational order. (B) Radial intensity profile obtained by integrating the diffraction intensities within the orange rings along the radial direction. (C) An example showing Gaussian fitting of a radial intensity peak.

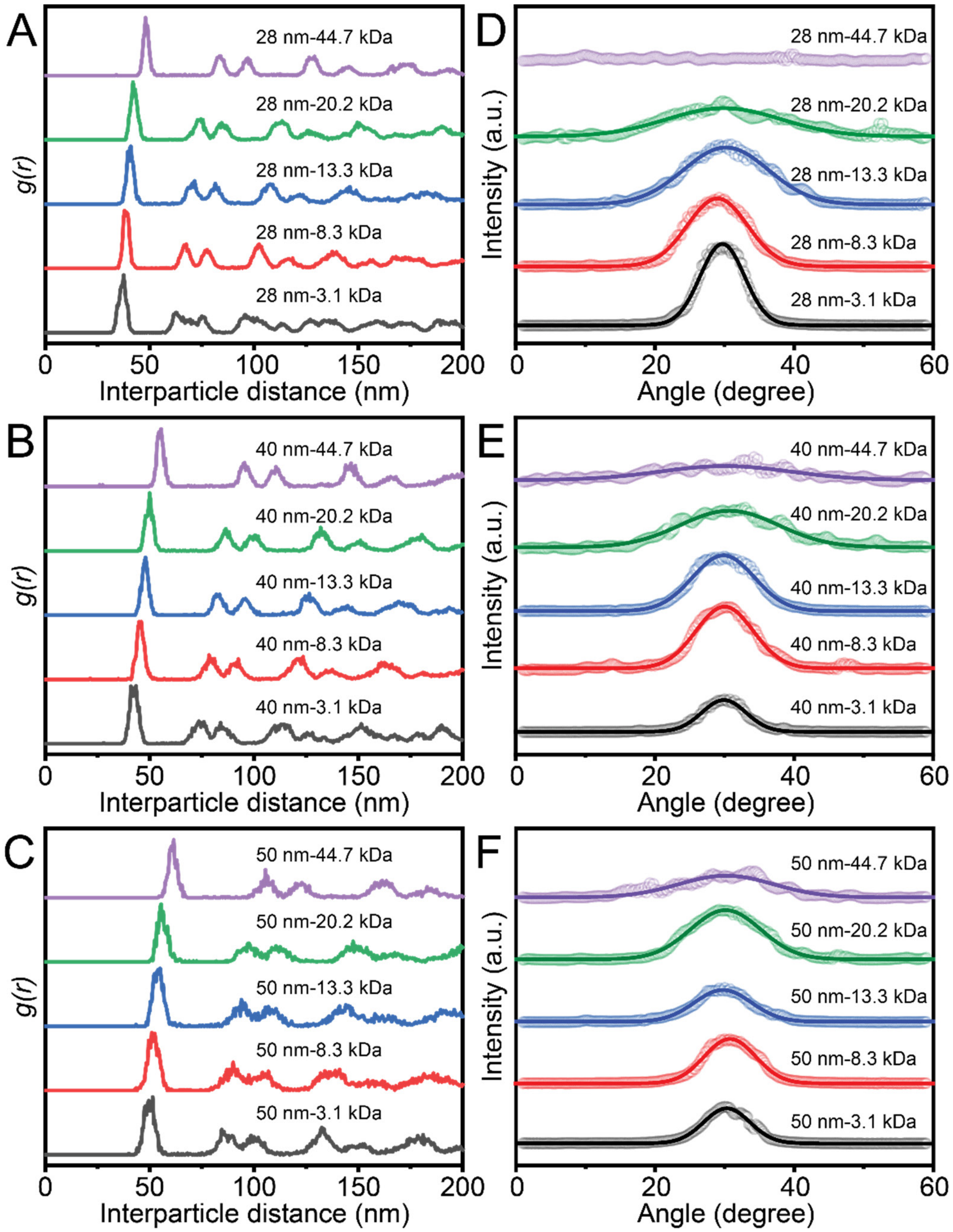

Fig. S10.

Evolution of translational and orientational order in 2D nanooctahedron superlattices as a function of nanooctahedron edge length and PS ligand molecular weight. (A-C) Radial distribution functions *g(r)* of various 2D superlattices calculated from the corresponding TEM images. (D-F) Radial intensity profiles obtained by radially integrating the $\{220\}_{MnO}$ diffraction intensities from the WAED patterns and corresponding Gaussian fits.

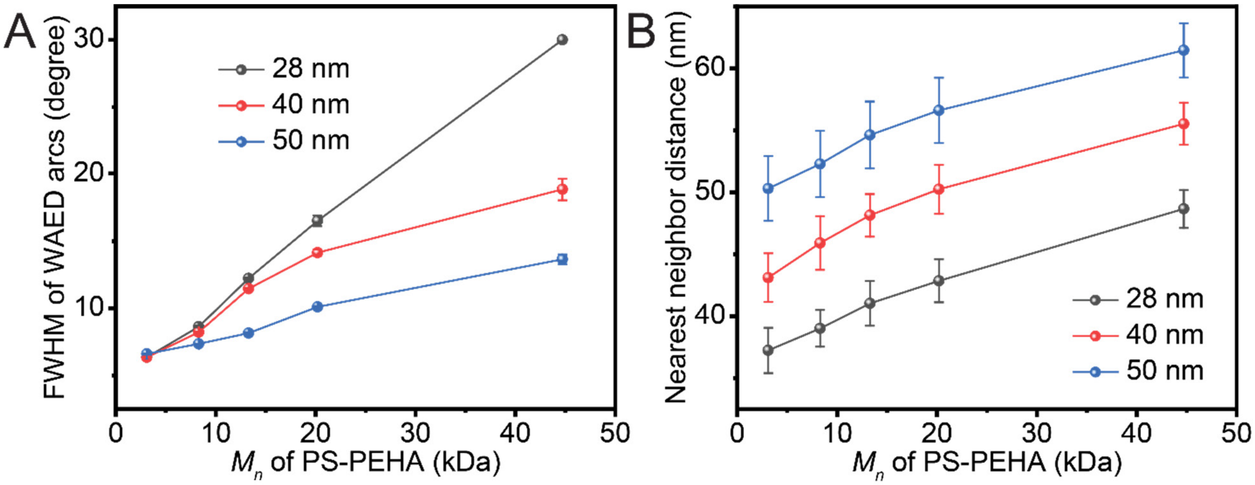

Fig. S11.

Structural evolution of 2D nanooctahedron superlattices as a function of nanooctahedron edge length and PS ligand molecular weight. (A) Plots of the full width at half maximum (FWHM) of the average radial intensities for the {220}$_{MnO}$ diffractions from WAED patterns of various 2D superlattices, shown as a function of nanooctahedron edge length and ligand molecular weight. (B) Plots of the nearest-neighbor interparticle distances as a function of nanooctahedron edge length and ligand molecular weight for various 2D superlattices.

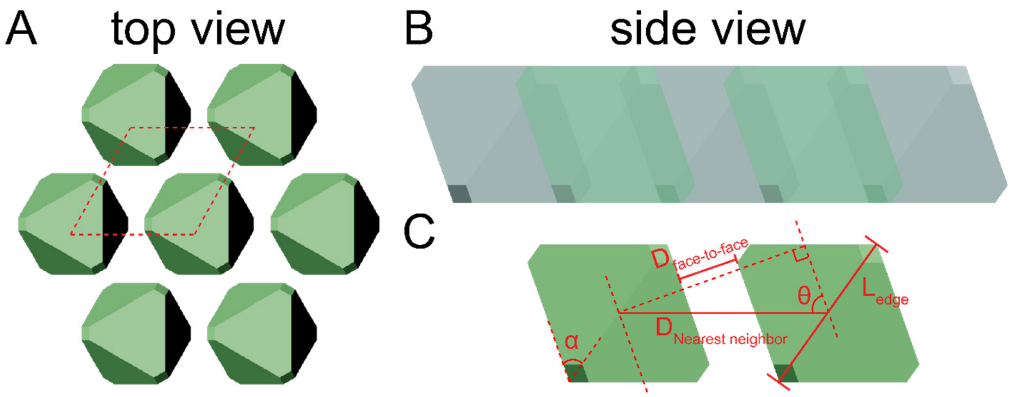

$$D_{face-to-face,\,2D} = \sin(\theta) \times D_{Nearest\ neighbor} - \sin(\alpha) \times L_{edge}$$

$$\theta = arcsin(\sqrt{8/9}) \qquad \alpha = arcsin(\sqrt{6/9})$$

Fig. S12.

Schematic illustration of face-to-face distance calculations in 2D superstructures. (a) Top view and (b) side view of the assembled structures. (c) Equations used for calculating face-to-face distances between adjacent nanooctahedra.

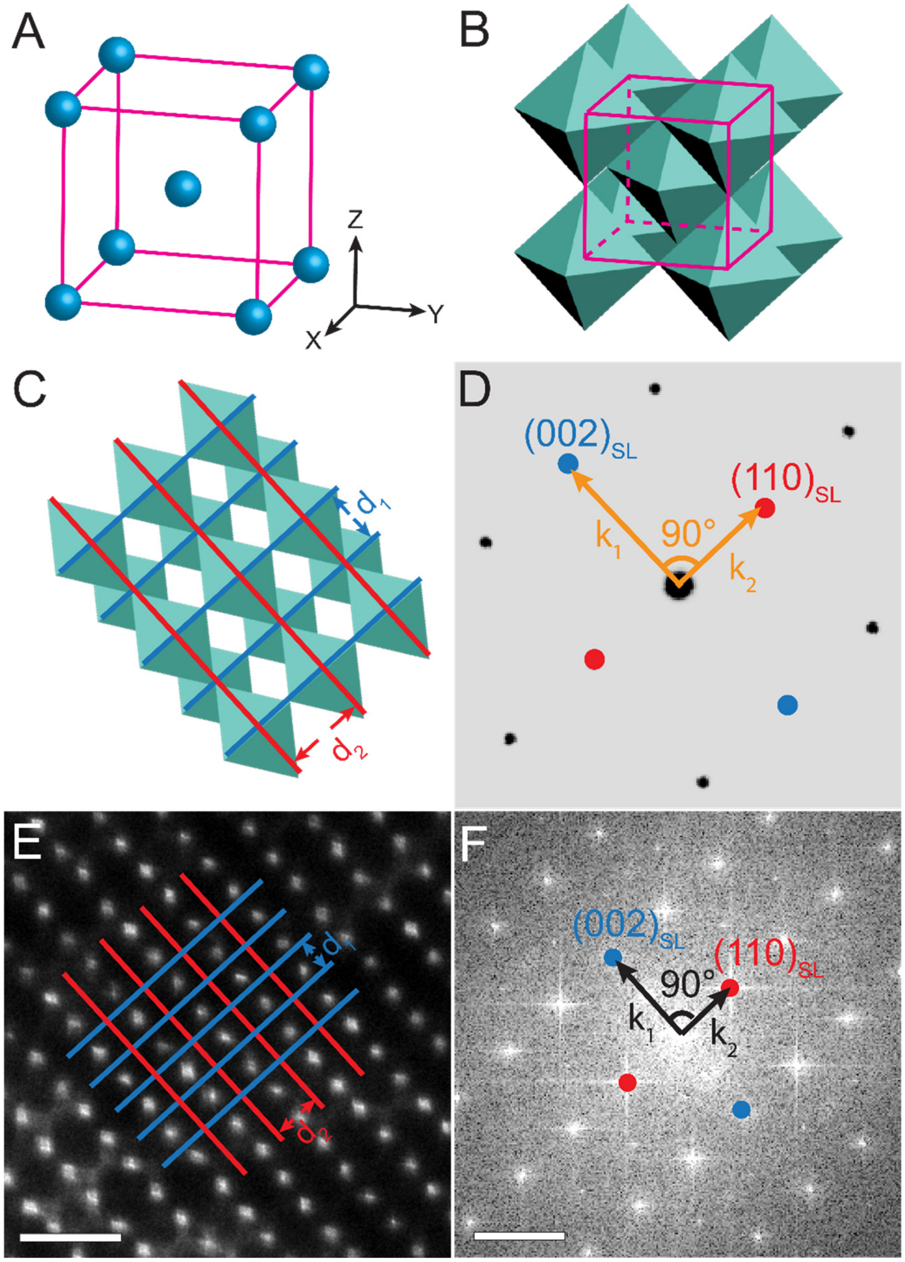

Fig. S13.

Structural analysis of the BCC superlattice. (A) Unit cell of a BCC lattice. (B) Structural model of a BCC lattice assembled from nanooctahedra. (C, D) Structural model of the $(110)_{SL}$ projection of a BCC lattice formed by nanooctahedra (C) and its corresponding FFT pattern (D). (E, F) Representative TEM image (E) and FFT pattern (F) of the BCC superlattice formed by 28 nm $MnO@Mn_3O_4$ nanooctahedra grafted with 8.3 kDa PS ligands. The ratio between the orthogonal k_1 and k_2 vectors is 1.41. Scale bars: (E) 50 nm, (F) 0.05 nm^{-1}.

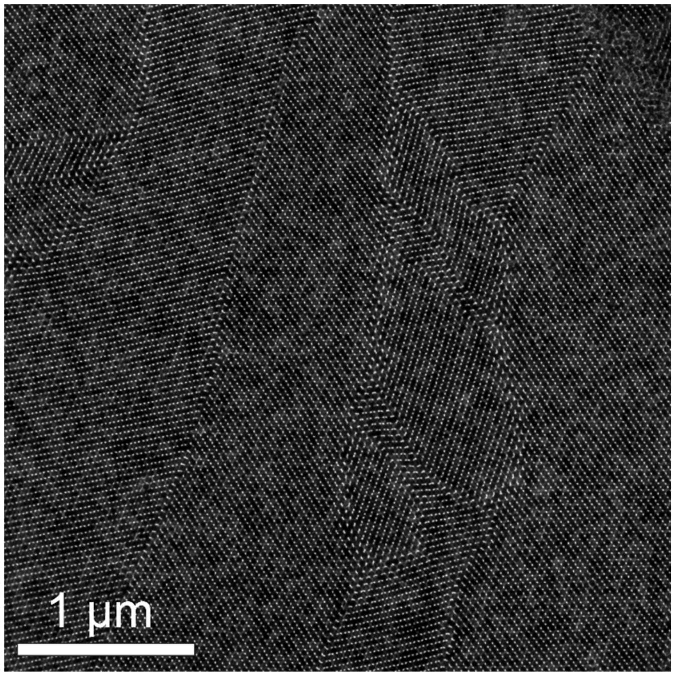

Fig. S14.

Low-magnification TEM image of the BCC superlattice assembled from 28 nm $MnO@Mn_3O_4$ nanooctahedra grafted with 8.3 kDa PS ligands.

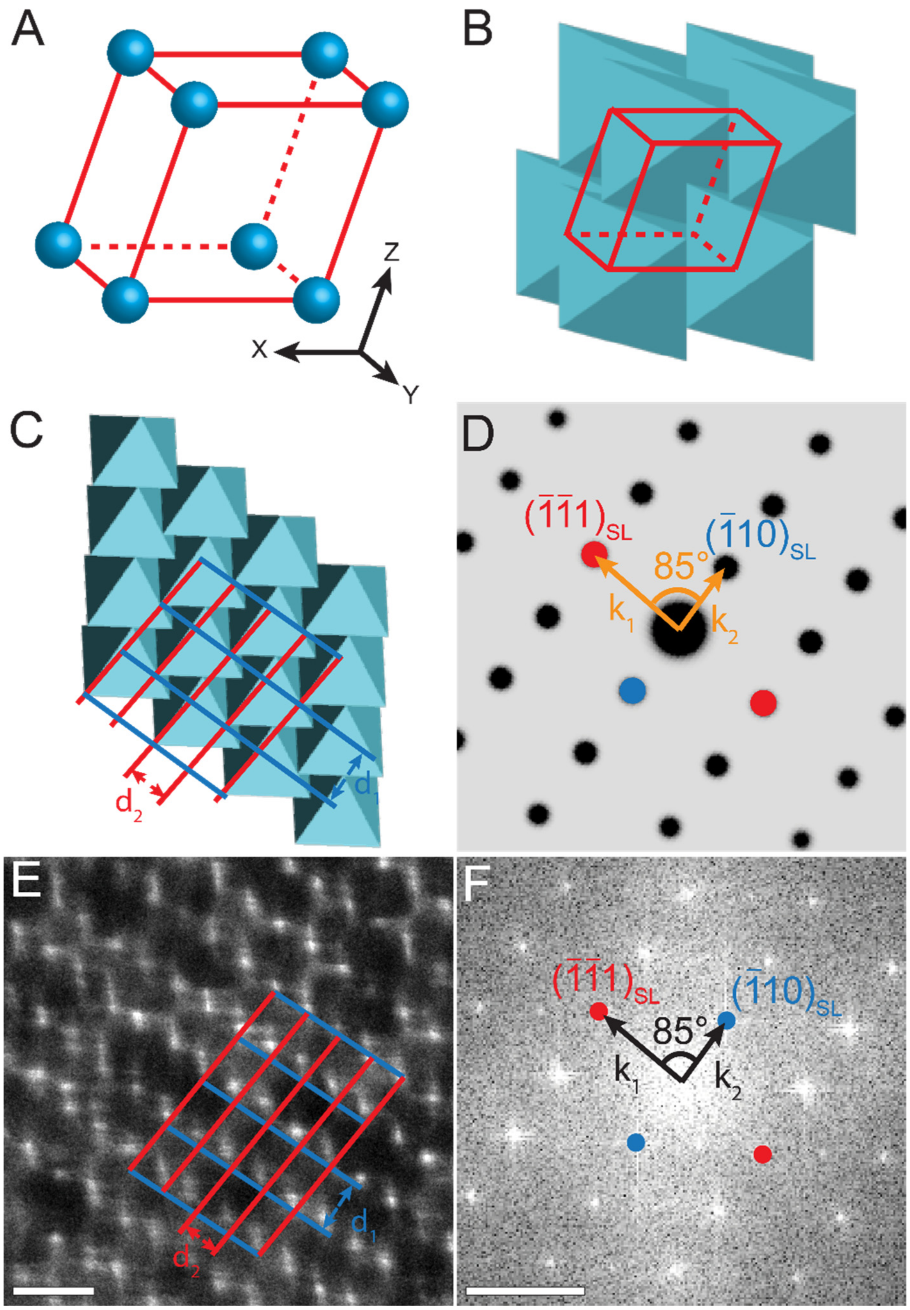

Fig. S15.

Structural analysis of the Minkowski superlattice. (A) Unit cell of a triclinic lattice. (B) Structural model of a Minkowski lattice assembled from nanooctahedra. (C, D) Structural model of the $(001)_{SL}$ projection of a Minkowski lattice formed by nanooctahedra (C) and its corresponding FFT pattern (D). (E, F) Representative TEM image (E) and FFT pattern (F) of the Minkowski superlattice formed by 40 nm $MnO@Mn_3O_4$ nanooctahedra grafted with 8.3 kDa PS ligands. The angle between the k_1 and k_2 vectors is 85°, with a ratio of 1.45 between their magnitudes. Scale bars: (E) 50 nm, (F) 0.05 nm^{-1}.

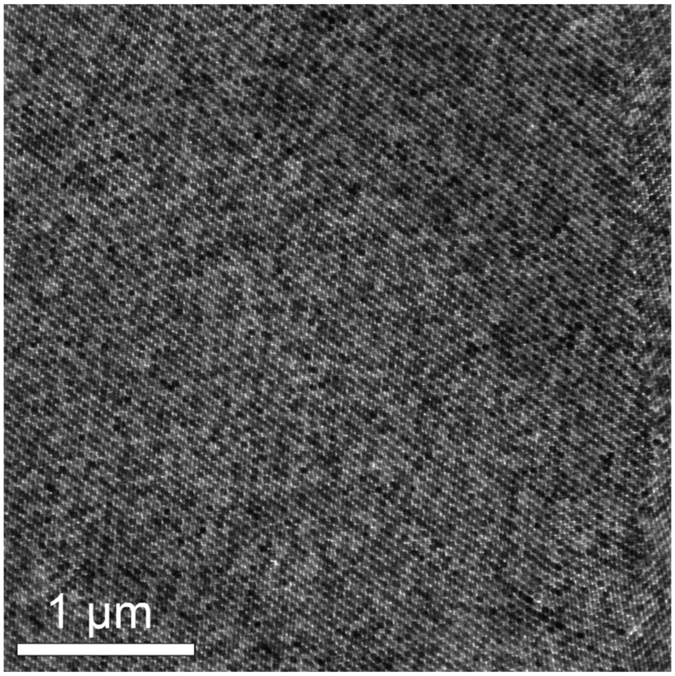

Fig. S16.

Low-magnification TEM image of the Minkowski superlattice assembled from 40 nm $MnO@Mn_3O_4$ nanooctahedra grafted with 8.3 kDa PS ligands.

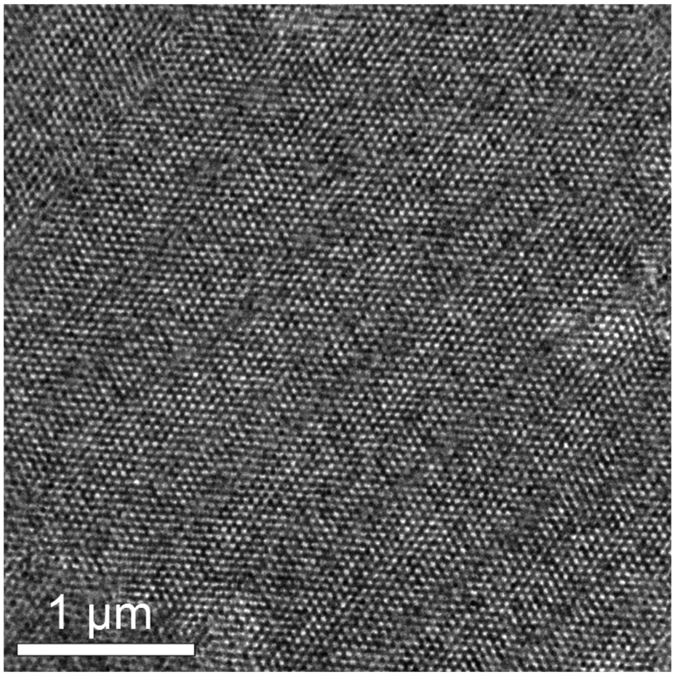

Fig. S17.

Low-magnification TEM image of the pHCP superlattice assembled from 28 nm $MnO@Mn_3O_4$ nanooctahedra grafted with 44.7 kDa PS ligands.

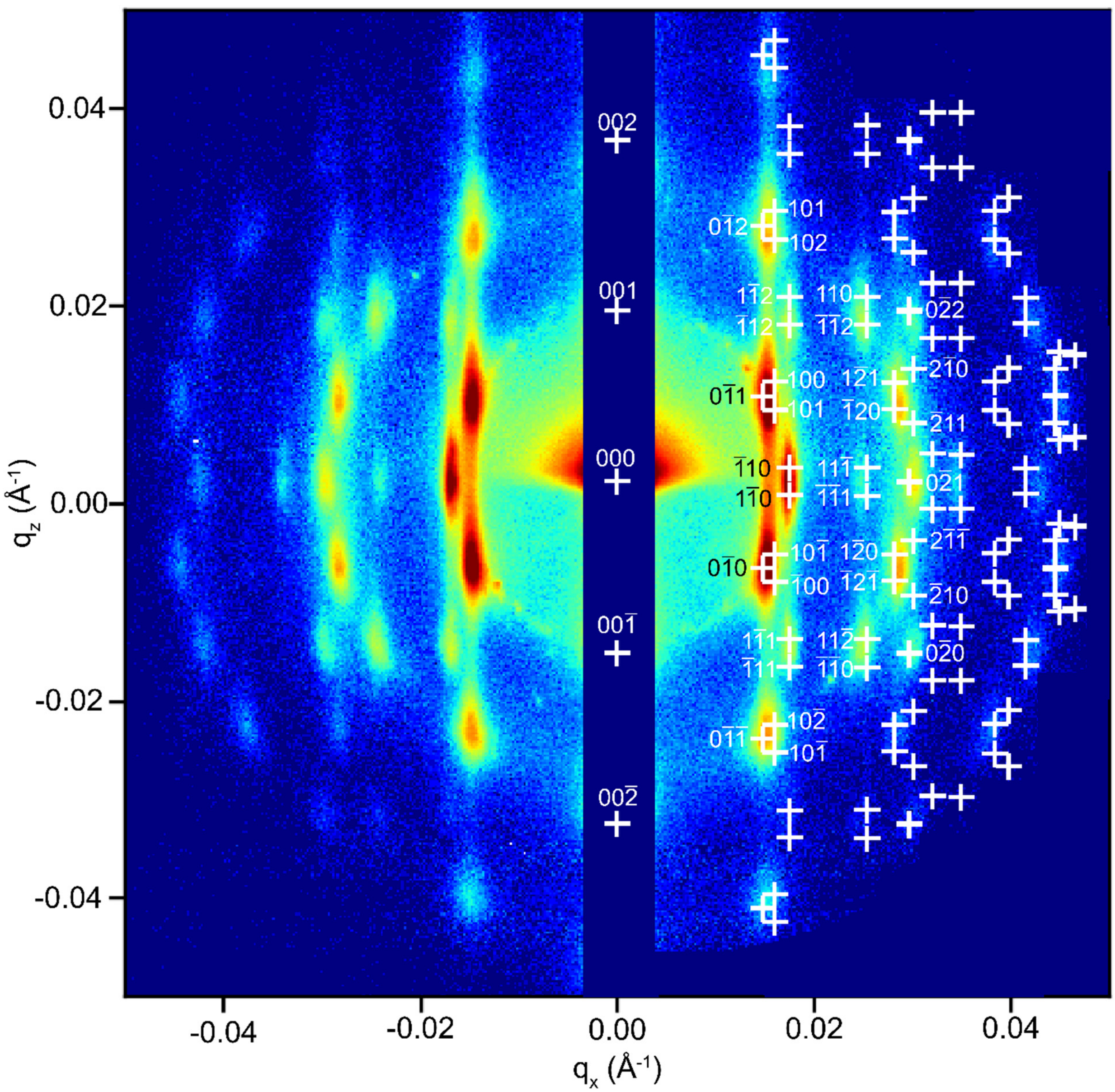

Fig. S18.

Representative GTSAXS pattern of the Minkowski superlattice assembled from 40 nm $MnO@Mn_3O_4$ nanooctahedra grafted with 8.3 kDa PS ligands, overlaid with the simulated scattering pattern and corresponding lattice plane indices.

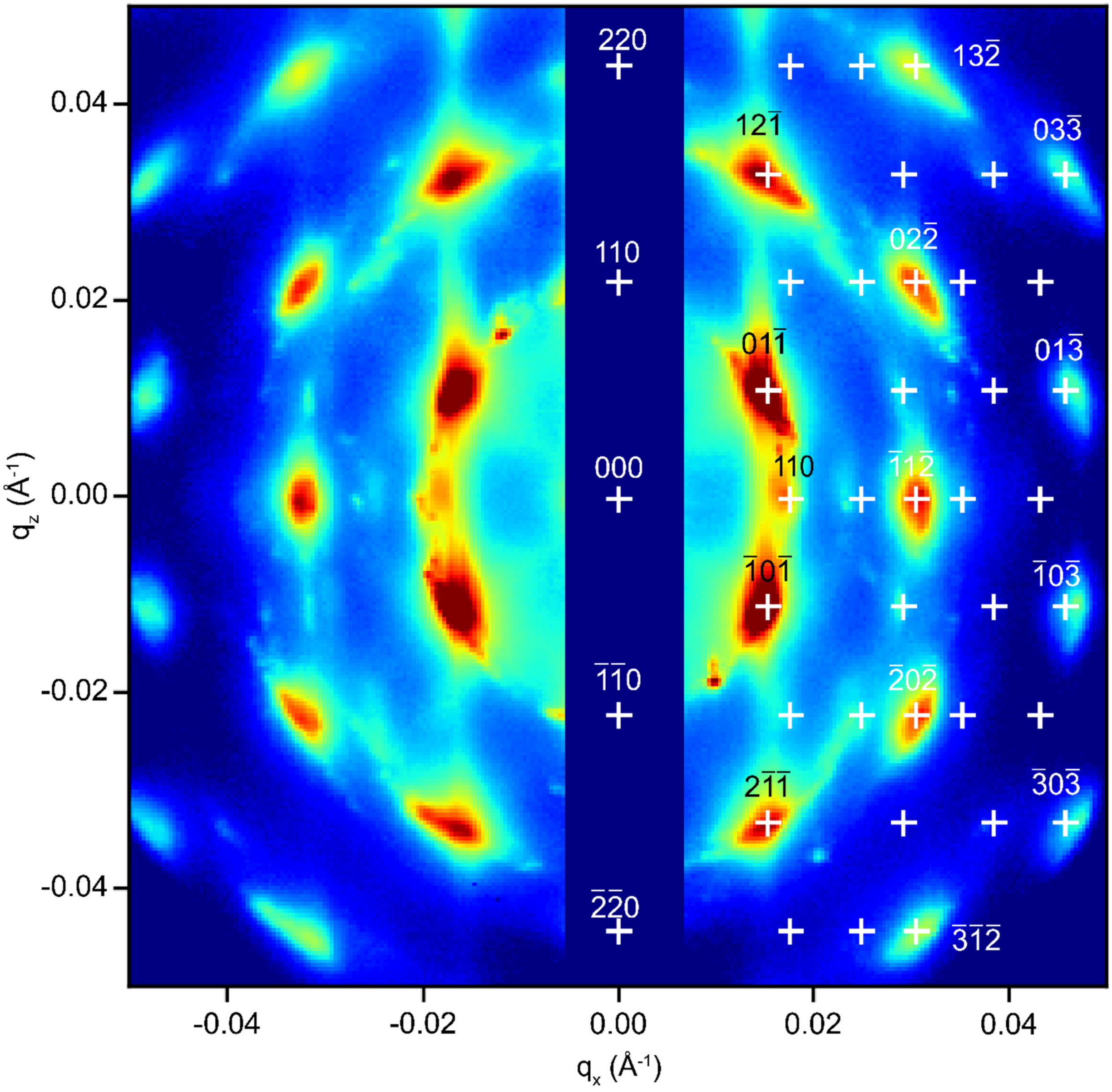

Fig. S19.

Representative GTSAXS pattern of the BCC superlattice assembled from 28 nm $MnO@Mn_3O_4$ nanooctahedra grafted with 8.3 kDa PS ligands, overlaid with the simulated scattering pattern and corresponding lattice plane indices.

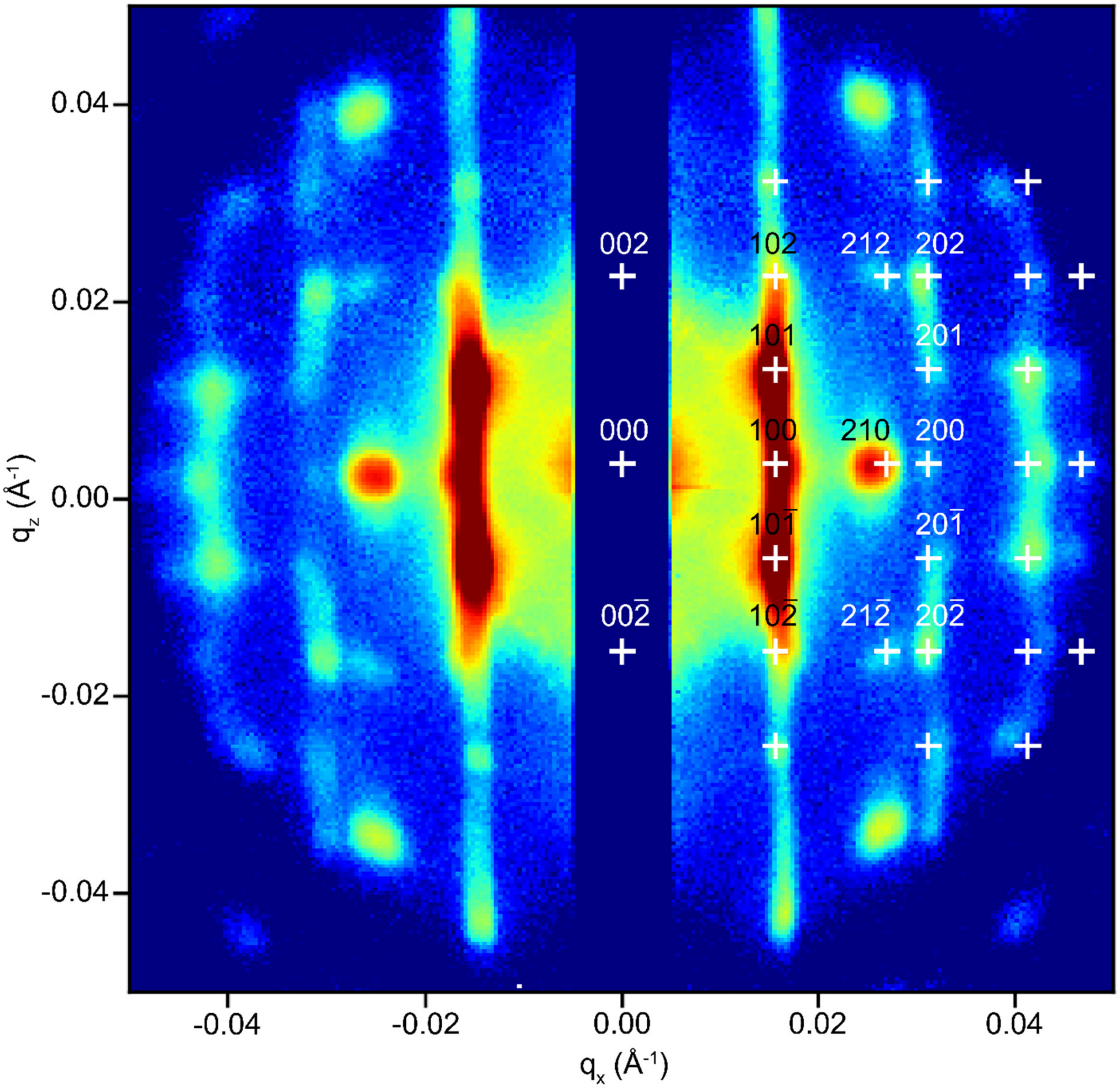

Fig. S20.

Representative GTSAXS pattern of the pHCP superlattice assembled from 28 nm $MnO@Mn_3O_4$ nanooctahedra grafted with 44.7 kDa PS ligands, overlaid with the simulated scattering pattern and corresponding lattice plane indices.

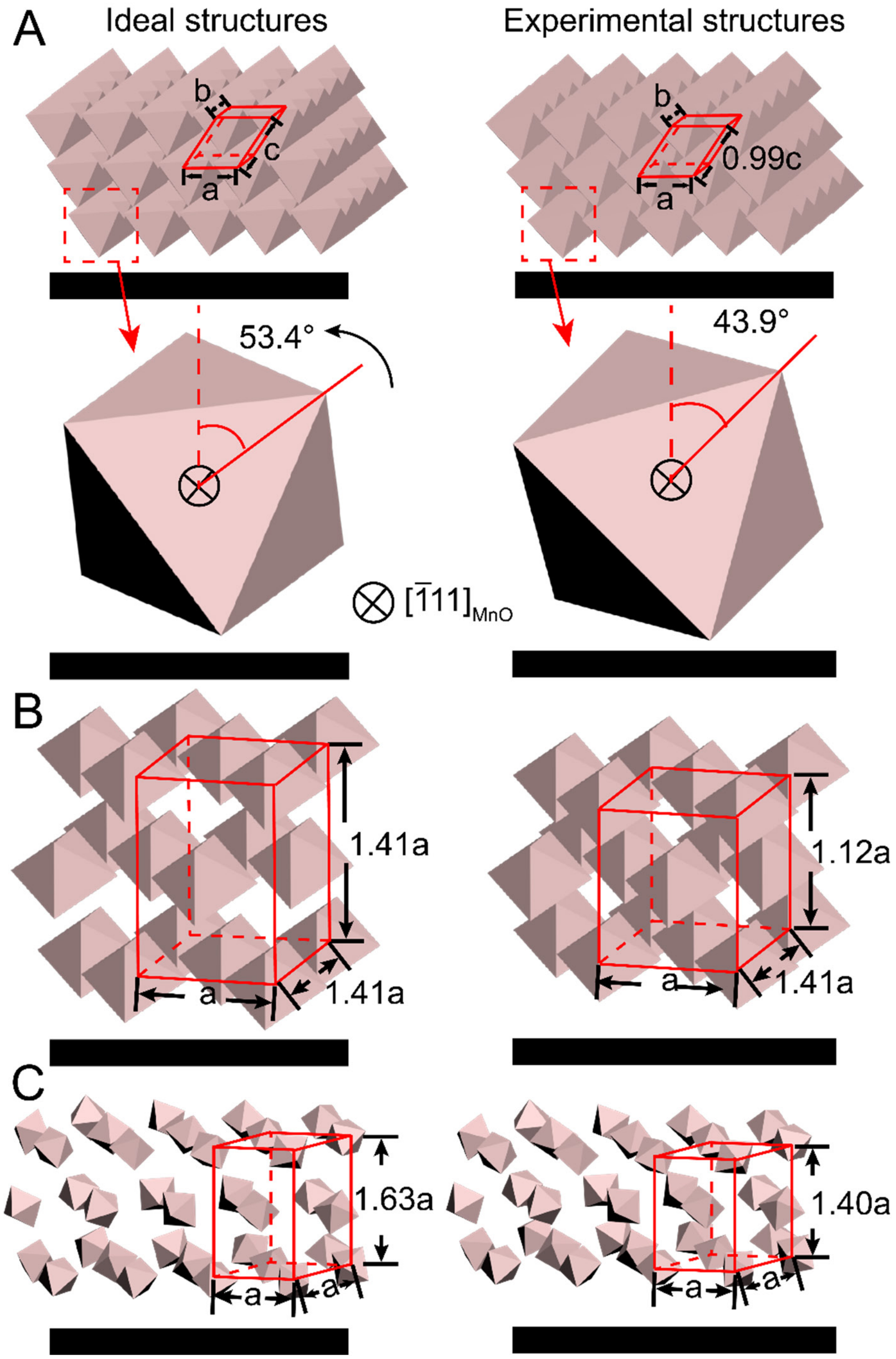

Fig. S21.

Schematic illustration of lattice contraction modes for the (A) Minkowski, (B) BCC, and (C) pHCP superlattices assembled from MnO@Mn_3O_4 nanooctahedra.

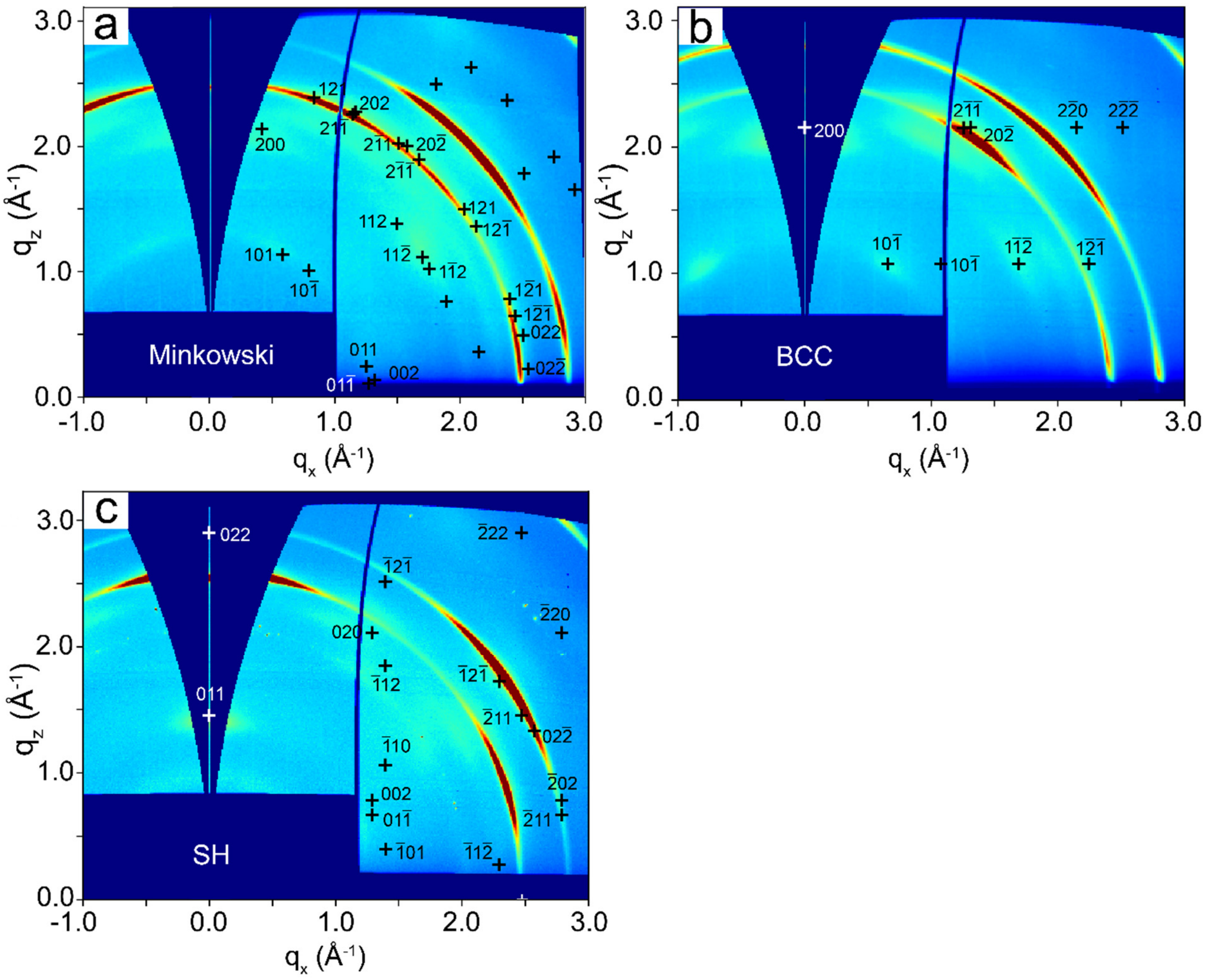

Fig. S22.

Representative GIWAXS patterns of the (A) Minkowski, (B) BCC, and (C) SH superlattices assembled from MnO@Mn_3O_4 nanooctahedra, overlaid with the simulated WAXS pattern of the Mn_3O_4 phase and corresponding lattice plane indices.

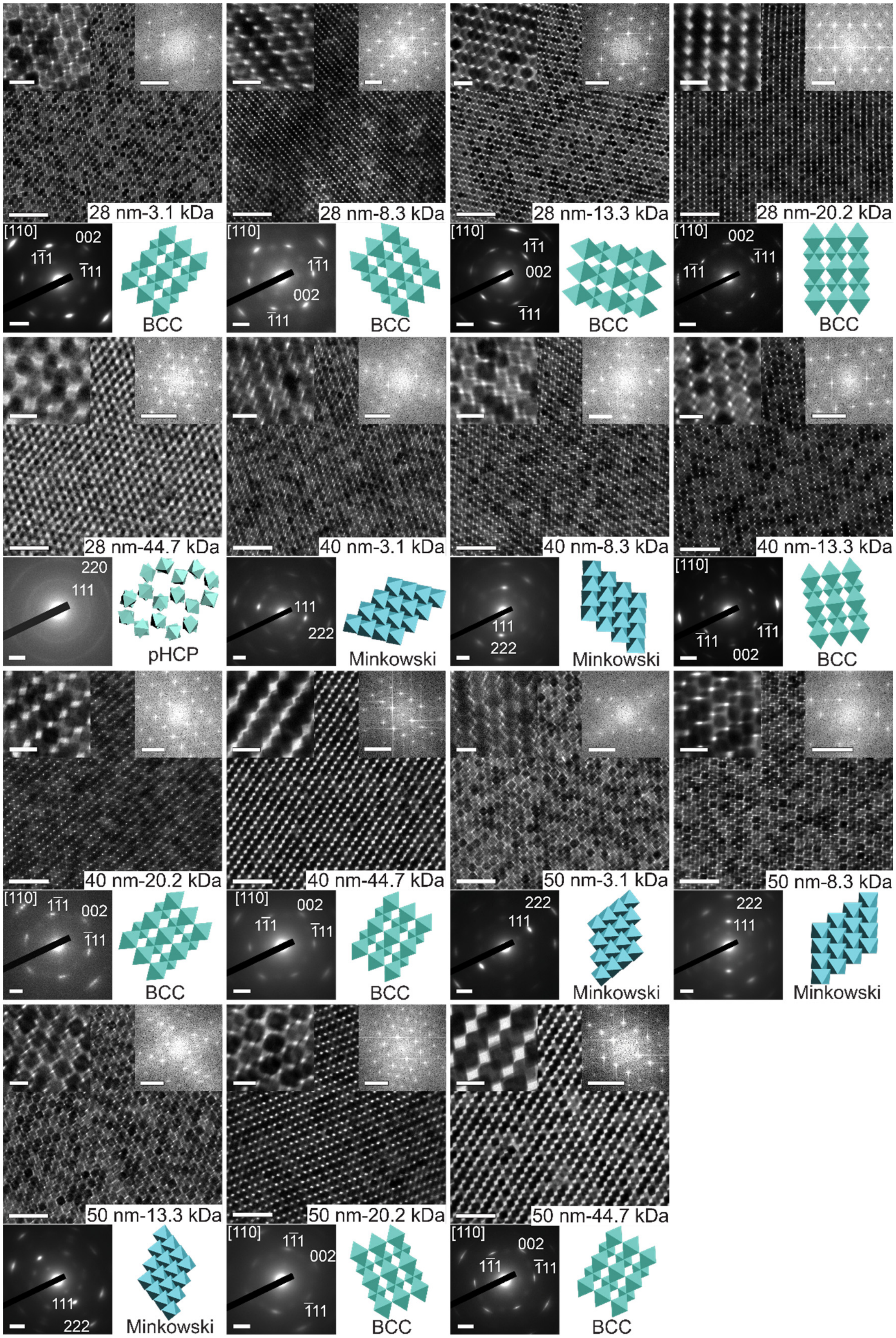

28 nm-3.1 kDa
[110]
002
$1\bar{1}1$
$\bar{1}11$
BCC
28 nm-8.3 kDa
[110]
$1\bar{1}1$
002
$\bar{1}11$
BCC
28 nm-13.3 kDa
[110]
$1\bar{1}1$
002
$\bar{1}11$
BCC
28 nm-20.2 kDa
[110]
002
$\bar{1}11$
$1\bar{1}1$
BCC
28 nm-44.7 kDa
220
111
pHCP
40 nm-3.1 kDa
111
222
Minkowski
40 nm-8.3 kDa
111
222
Minkowski
40 nm-13.3 kDa
[110]
$\bar{1}11$
$1\bar{1}1$
002
BCC
40 nm-20.2 kDa
[110]
$1\bar{1}1$
002
$\bar{1}11$
BCC
40 nm-44.7 kDa
[110]
002
$1\bar{1}1$
$\bar{1}11$
BCC
50 nm-3.1 kDa
222
111
Minkowski
50 nm-8.3 kDa
222
111
Minkowski
50 nm-13.3 kDa
111
222
Minkowski
50 nm-20.2 kDa
[110]
$1\bar{1}1$
002
$\bar{1}11$
BCC
50 nm-44.7 kDa
[110]
002
$1\bar{1}1$
$\bar{1}11$
BCC

Fig. S23.

Superstructural phase transitions in 3D superlattices assembled from PS-grafted MnO@Mn_3O_4 nanooctahedra. Each panel displays a low-magnification TEM image (top), a high-magnification TEM image (upper left inset), the corresponding FFT pattern (upper right inset), the WAED pattern (bottom left inset), and a structural model (bottom right inset) for various 3D superlattices assembled from PS-grafted MnO@Mn_3O_4 nanooctahedra. Scale bars: 200 nm for low-magnification TEM images, 50 nm for high-magnification TEM images, 2 nm^{-1} for WAED patterns, and 0.05 nm^{-1} for FFT patterns.

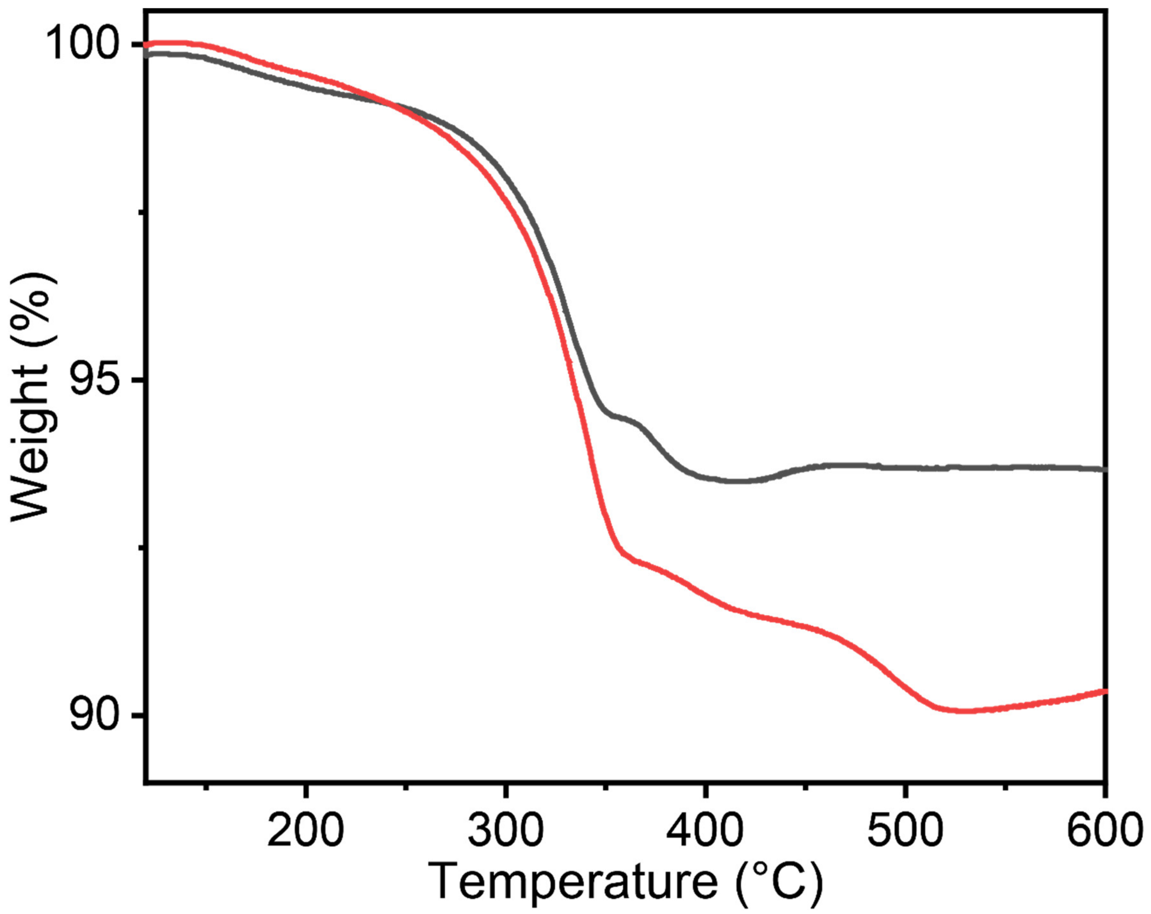

Fig. S24.

TGA results of 50 nm $MnO@Mn_3O_4$ nanooctahedra grafted with 8.3 kDa PS ligands at grafting densities of σ = 0.26 chains/nm^2 (black) and (b) σ = 0.38 chains/nm^2 (red).

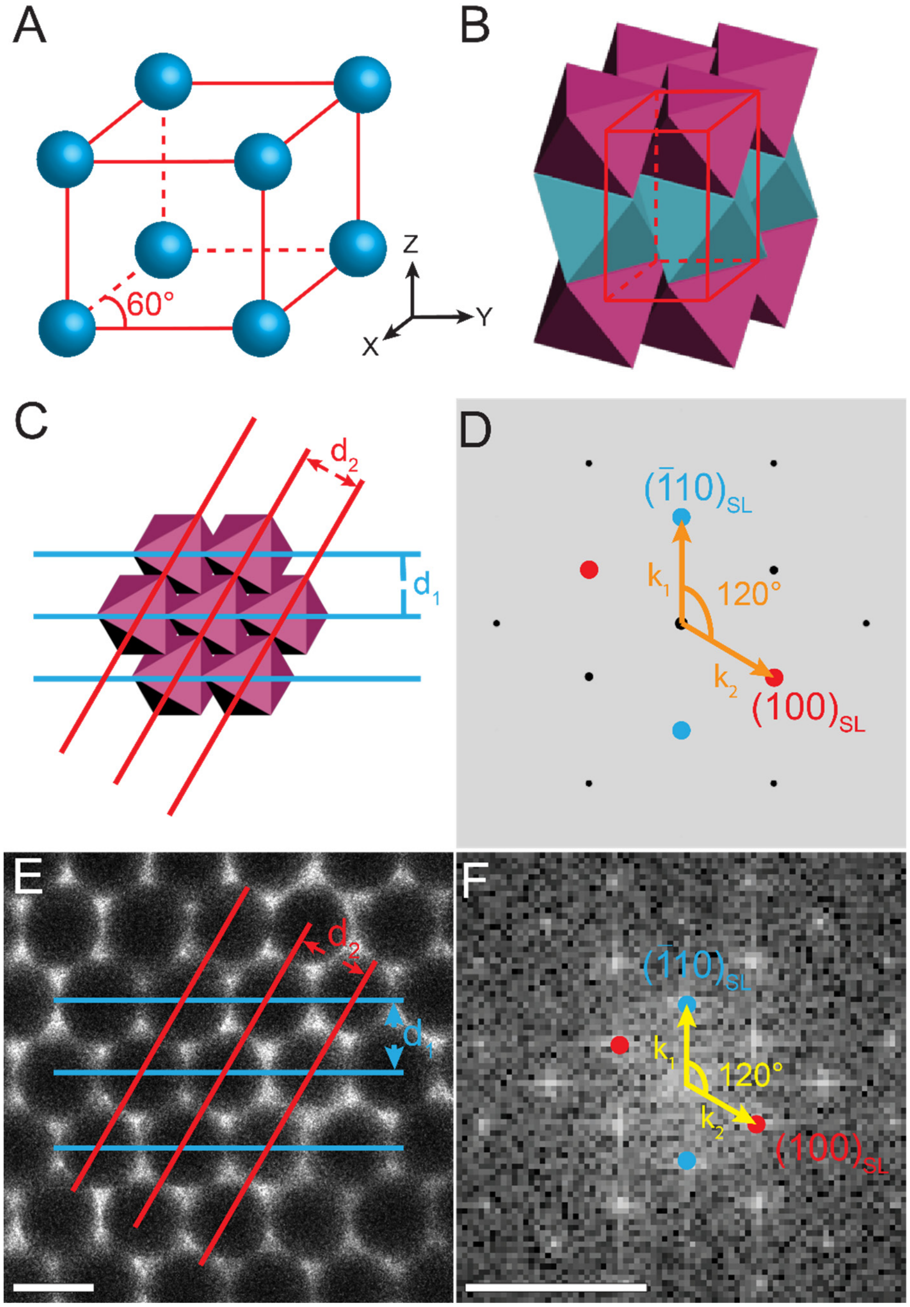

Fig. S25.

Structural analysis of the SH superlattice. (A) Unit cell of an SH lattice. (B) Structural model of an SH lattice assembled from nanooctahedra. (C-D) Structural model of the $(0001)_{SL}$ projection of an SH lattice formed by nanooctahedra (C) and its corresponding FFT pattern (D). (E-F) Representative TEM image (E) and FFT pattern of the SH superlattice formed by 50 nm $MnO@Mn_3O_4$ nanooctahedra grafted with 8.3 kDa PS ligands. The angle between the k_1 and k_2 vectors is 60°, with their magnitudes being equal. Scale bars: (E) 50 nm, (F) 0.05 nm^{-1}.

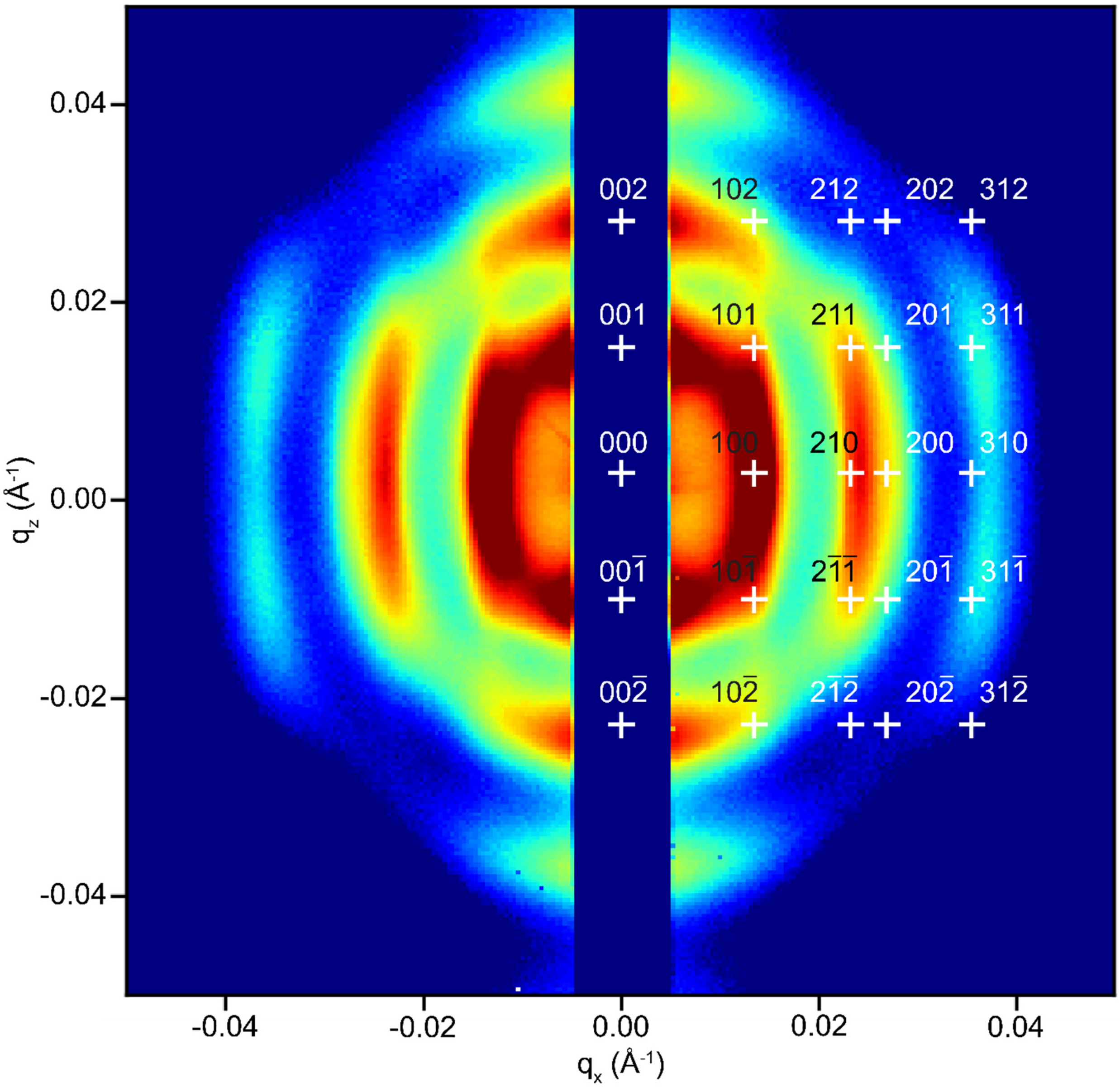

Fig. S26.

Representative GTSAXS pattern of the SH superlattice assembled from 50 nm $MnO@Mn_3O_4$ nanooctahedra grafted with 8.3 kDa PS ligands (σ = 0.38 chains/nm^2), overlaid with the simulated scattering pattern and corresponding lattice plane indices.

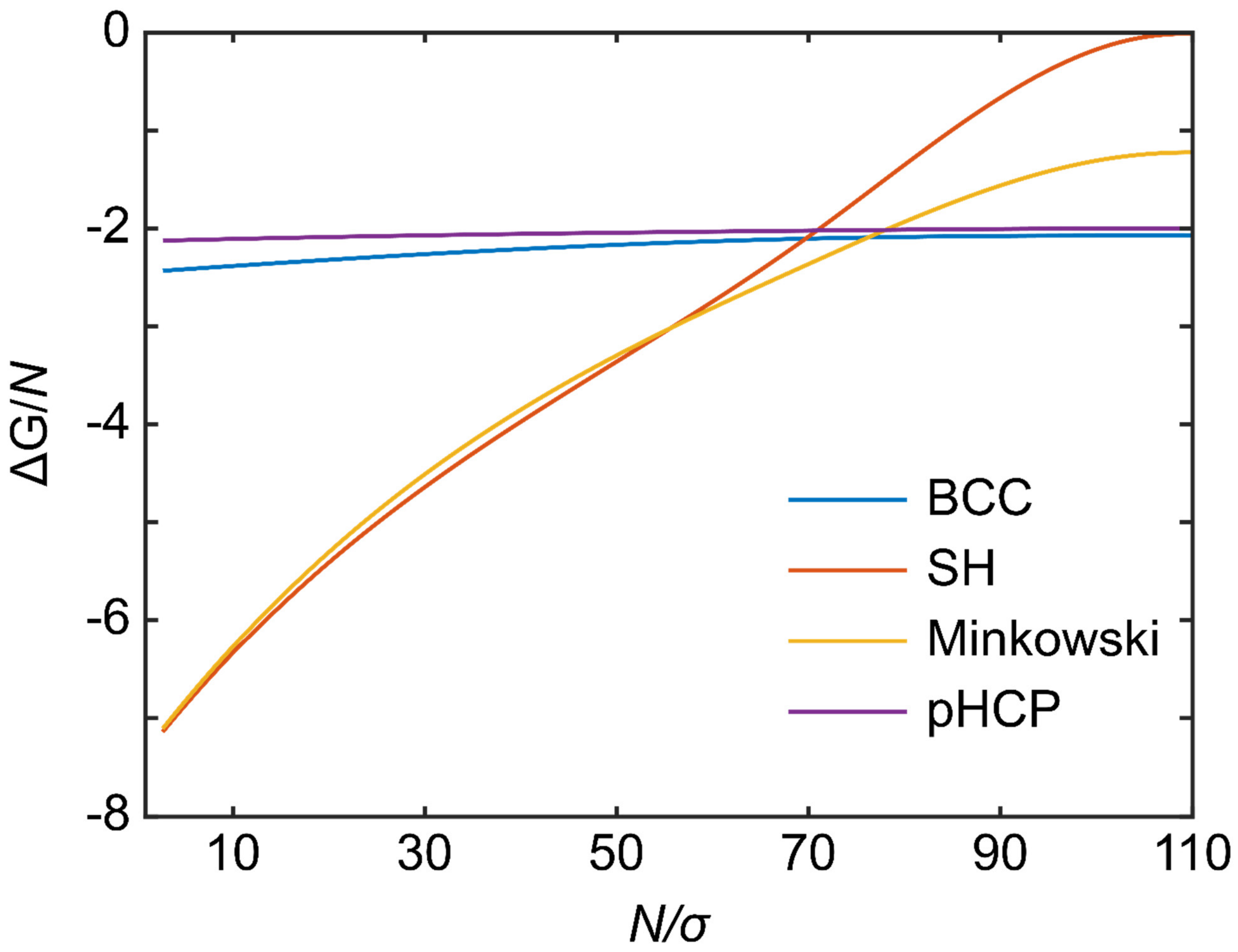

Fig. S27.

Plots of the calculated excess free energy of formation per NC as a function of the ratio of polymer chain length (*N*) and polymer grafting density (*σ*). The results indicate significant differences in the BCC and pHCP relative to SH and Minkowski lattices, reflecting the shift in the directional interactions mediated by the corona morphology for each NC. Small energy differences are observed between Minkowski and SH, suggesting that kinetic effects may influence the experimentally observed phase behaviors.

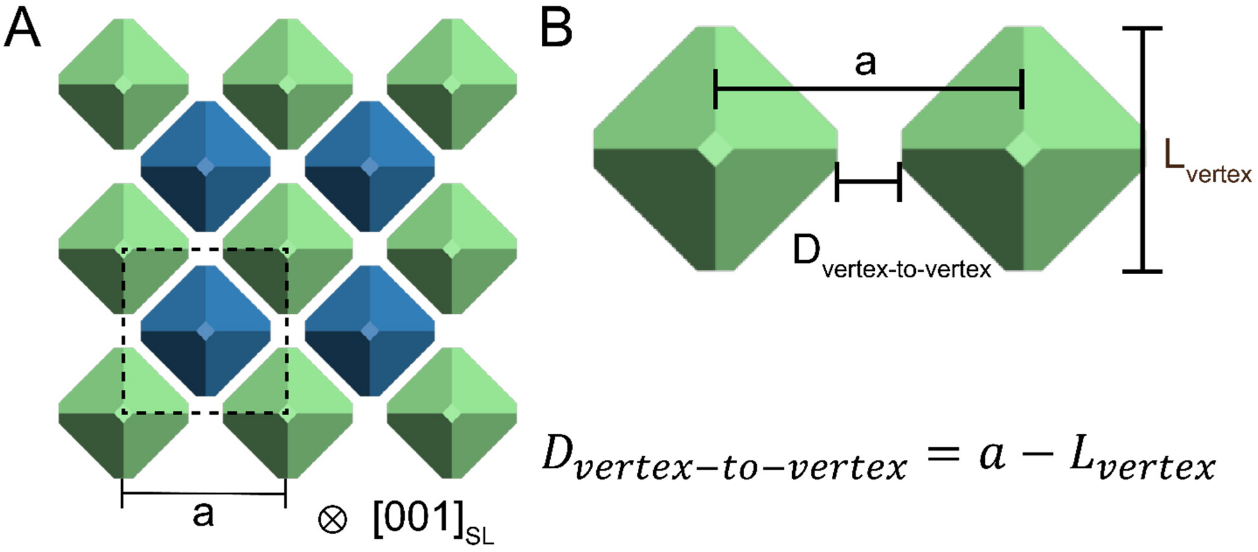

Fig. S28.

Schematic illustration of vertex-to-vertex distance calculations in 3D BCC superstructures. (a) Projection along the [001]SL zone axis of the BCC lattice. (b) Equation used for calculating the vertex-to-vertex distance between adjacent nanooctahedra within BCC superlattices.

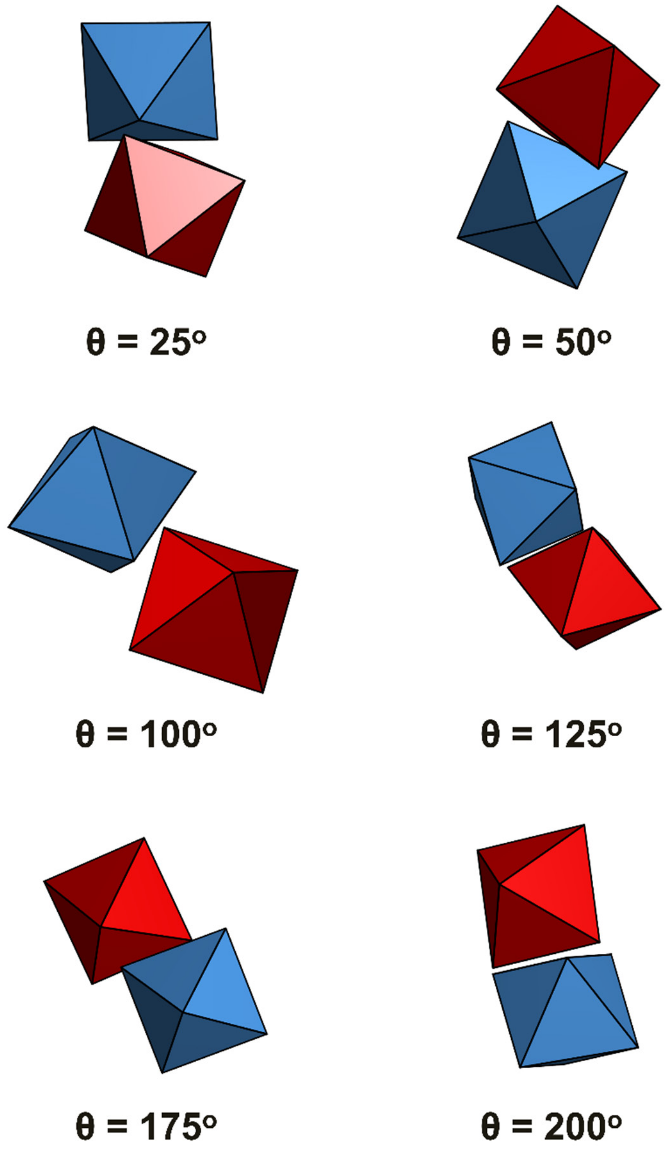

Fig. S29.

Simulation snapshots showing particle pair configurations between octahedra at various relative angles. These angles map to the angular distribution plots shown in Figure 5e, h, k, and n of the main text.

Table S1.

Summary of reaction parameters for PS-Br synthesis.

M_n of PS-PEHA (kDa)	Molar ratio					Time (h)	Toluene (mL)	Monomer conversion (%)	M_n of PS-Br (Da)	M_w/M_n of PS-Br
	Styrene	EBIB	$CuBr_2$	Me_6TREN	$Sn(EH)_2$					
3.1	30	1	0.01	0.1	0.1	22	25	66.3	2,981	1.20
8.3	100	1	0.01	0.1	0.1	18	0	61.2	8,050	1.05
13.3	200	1	0.01	0.1	0.1	25	0	56.5	13,091	1.12
20.2	400	1	0.01	0.1	0.1	17	0	55.0	19,858	1.10
44.7	600	1	0.01	0.1	0.1	21	0	52.0	44,410	1.11

Table S2.

Summary of experimental parameters for the polymer ligand exchange process and measured polymer grafting densities from TGA.

Edge length of nanooctahedra (nm)	M_n of PS-PEHA (kDa)	Feeding grafting density (chains/nm^2)	Grafting density measured using TGA (chains/nm^2)
28	3.1	7.5	0.58
	8.3		0.42
	13.3		0.33
	20.2		0.33
	44.7		0.28
40	3.1		0.57
	8.3		0.35
	13.3		0.33
	20.2		0.26
	44.7		0.20
50	3.1		0.32
	8.3		0.26
	13.3		0.22
	20.2		0.17
	44.7		0.11
	8.3	30	0.38

Table S3.

Summary of normalized face-to-face distances between adjacent nanooctahedra in 2D superstructures, scaled by polymer molecular weight and grafting density.

M_n of PS-PEHA (kDa)	$D_{\text{face-to-face, 2D}}$ (nm)	σ (chains/nm^2)	$D_{\text{face-to-face, 2D}}$/ ($M_n^{0.5}\times\sigma^{0.25}$)
3.1	7.98	0.57	5.22
8.3	10.62	0.35	4.79
13.3	12.74	0.33	4.61
20.2	14.71	0.26	4.58
44.7	19.69	0.20	4.40

Table S4.

Summary of lattice parameters of various 3D superlattices assembled from $MnO@Mn_3O_4$ nanooctahedra calculated based on TEM images.

Nanocrystal building blocks	Lattice type	a (nm)	b (nm)	c (nm)	α (°)	β (°)	γ (°)
28 nm-3.1 kDa	BCC	34.22	34.22	34.22	90	90	90
28 nm-8.3 kDa	BCC	36.63	36.63	36.63	90	90	90
28 nm-13.3 kDa	BCC	40.98	45.53	45.53	90	90	90
28 nm-20.2 kDa	BCC	42.41	47.90	47.90	90	90	90
28 nm-44.7 kDa	pHCP	47.75	47.75	77.97	90	90	120
40 nm-3.1 kDa	Minkowski	40.95	44.23	44.23	108	111	111
40 nm-8.3 kDa	Minkowski	42.31	45.70	45.70	108	111	111
40 nm-13.3 kDa	BCC	53.20	53.20	53.20	90	90	90
40 nm-20.2 kDa	BCC	55.25	55.25	55.25	90	90	90
40 nm-44.7 kDa	BCC	63.96	63.96	63.96	90	90	90
50 nm-3.1 kDa	Minkowski	44.56	48.13	48.13	108	111	111
50 nm-8.3 kDa 0.26 chains/nm^2	Minkowski	45.87	49.55	49.55	108	111	111
50 nm-13.3 kDa	Minkowski	49.45	53.41	53.41	108	111	111
50 nm-20.2 kDa	BCC	62.26	62.26	62.26	90	90	90
50 nm-44.7 kDa	BCC	68.35	68.35	68.35	90	90	90
50 nm-8.3 kDa, 0.38 chains/nm^2	SH	53.96	53.96	101.74	90	90	120

Table S5.

Summary of normalized vertex-to-vertex distances between adjacent nanooctahedra in BCC superstructures, scaled by polymer molecular weight and grafting density.

M_n of PS-PEHA (kDa)	$D_{vertex\text{-}to\text{-}vertex}$ (nm)	σ (chains/nm^2)	$D_{vertex\text{-}to\text{-}vertex}/(M_n^{0.5}\times\sigma^{0.25})$
13.3	1.68	0.33	0.61
20.2	3.77	0.26	1.17
44.7	12.48	0.20	2.79

Supplementary Movie Captions

Movie S1.
TEM tomography reconstruction of a representative $MnO@Mn_3O_4$ nanooctahedron.

Movie S2.
Computer simulation of nanooctahedra self-assembly into a Minkowski superlattice.

Movie S3.
Computer simulation of nanooctahedra self-assembly into a BCC superlattice.

Movie S4.
Computer simulation of nanooctahedra self-assembly into a pHCP superlattice.

Movie S5
Computer simulation of nanooctahedra self-assembly into an SH superlattice.

REFERENCES AND NOTES

1. M. A. Boles, M. Engel, D. V. Talapin, Self-assembly of colloidal nanocrystals: From intricate structures to functional materials. *Chem. Rev.* **116**, 11220–11289 (2016).

2. Z. Li, Q. Fan, Y. Yin, Colloidal self-assembly approaches to smart nanostructured materials. *Chem. Rev.* **122**, 4976–5067 (2022).

3. Y. Zhong, V. R. Allen, J. Chen, Y. Wang, X. Ye, Multistep crystallization of dynamic nanoparticle superlattices in nonaqueous solutions. *J. Am. Chem. Soc.* **144**, 14915–14922 (2022).

4. G. Singh, H. Chan, A. Baskin, E. Gelman, N. Repnin, P. Král, R. Klajn, Self-assembly of magnetite nanocubes into helical superstructures. *Science* **345**, 1149–1153 (2014).

5. Y. Nagaoka, R. Tan, R. Li, H. Zhu, D. Eggert, Y. A. Wu, Y. Liu, Z. Wang, O. Chen, Superstructures generated from truncated tetrahedral quantum dots. *Nature* **561**, 378–382 (2018).

6. C. A. Batista, R. G. Larson, N. A. Kotov, Nonadditivity of nanoparticle interactions. *Science* **350**, 1242477 (2015).

7. J. Henzie, M. Grunwald, A. Widmer-Cooper, P. L. Geissler, P. Yang, Self-assembly of uniform polyhedral silver nanocrystals into densest packings and exotic superlattices. *Nat. Mater.* **11**, 131–137 (2011).

8. Y. Wang, J. Chen, Y. Zhong, S. Jeong, R. Li, X. Ye, Structural diversity in dimension-controlled assemblies of tetrahedral gold nanocrystals. *J. Am. Chem. Soc.* **144**, 13538–13546 (2022).

9. S. Zhou, J. Li, J. Lu, H. Liu, J. Y. Kim, A. Kim, L. Yao, C. Liu, C. Qian, Z. D. Hood, X. Lin, W. Chen, T. E. Gage, I. Arslan, A. Travesset, K. Sun, N. A. Kotov, Q. Chen, Chiral assemblies of pinwheel superlattices on substrates. *Nature* **612**, 259–265 (2022).

10. Z. Cheng, M. R. Jones, Assembly of planar chiral superlattices from achiral building blocks. *Nat. Commun.* **13**, 4207 (2022).

11. H. Lin, S. Lee, L. Sun, M. Spellings, M. Engel, S. C. Glotzer, C. A. Mirkin, Clathrate colloidal crystals. *Science* **355**, 931–935 (2017).

12. J. J. Choi, C. R. Bealing, K. Bian, K. J. Hughes, W. Zhang, D. M. Smilgies, R. G. Hennig, J. R. Engstrom, T. Hanrath, Controlling nanocrystal superlattice symmetry and shape-anisotropic interactions through variable ligand surface coverage. *J. Am. Chem. Soc.* **133**, 3131–3138 (2011).

13. X. Ye, J. E. Collins, Y. Kang, J. Chen, D. T. Chen, A. G. Yodh, C. B. Murray, Morphologically controlled synthesis of colloidal upconversion nanophosphors and their shape-directed self-assembly. *Proc. Natl. Acad. Sci. U.S.A.* **107**, 22430–22435 (2010).

14. W. Zhou, Y. Li, B. E. Partridge, C. A. Mirkin, Engineering anisotropy into organized nanoscale matter. *Chem. Rev.* **124**, 11063–11107 (2024).

15. Y. Zhong, T. C. Moore, T. Dwyer, A. Butrum-Griffith, V. R. Allen, J. Chen, Y. Wang, F. Cheng, S. C. Glotzer, X. Ye, Engineering and direct imaging of nanocube self-assembly pathways. *Nat. Chem. Eng.* **1**, 532–541 (2024).

16. Y. Liu, M. Klement, Y. Wang, Y. Zhong, B. Zhu, J. Chen, M. Engel, X. Ye, Macromolecular ligand engineering for programmable nanoprism assembly. *J. Am. Chem. Soc.* **143**, 16163–16172 (2021).

17. Y. Wang, J. Chen, C. Zhu, B. Zhu, S. Jeong, Y. Yi, Y. Liu, J. Fiadorwu, P. He, X. Ye, Kinetically controlled self-assembly of binary polymer-grafted nanocrystals into ordered superstructures via solvent vapor annealing. *Nano Lett.* **21**, 5053–5059 (2021).

18. X. Ye, C. Zhu, P. Ercius, S. N. Raja, B. He, M. R. Jones, M. R. Hauwiller, Y. Liu, T. Xu, A. P. Alivisatos, Structural diversity in binary superlattices self-assembled from polymer-grafted nanocrystals. *Nat. Commun.* **6**, 10052 (2015).

19. M. N. O'Brien, M. Girard, H. X. Lin, J. A. Millan, M. Olvera de la Cruz, B. Lee, C. A. Mirkin, Exploring the zone of anisotropy and broken symmetries in DNA-mediated nanoparticle crystallization. *Proc. Natl. Acad. Sci. U.S.A.* **113**, 10485–10490 (2016).

20. F. Lu, T. Vo, Y. Zhang, A. Frenkel, K. G. Yager, S. Kumar, O. Gang, Unusual packing of soft-shelled nanocubes. *Sci. Adv.* **5**, eaaw2399 (2019).

21. K. J. Si, Y. Chen, Q. Shi, W. Cheng, Nanoparticle superlattices: The roles of soft ligands. *Adv. Sci.* **5**, 1700179 (2018).

22. Y. H. Lee, W. Shi, Y. Yang, Y.-C. Kao, H. K. Lee, R. Chu, Y. L. Pang, C. L. Lay, S. Li, X. Y. Ling, Modulating orientational order to organize polyhedral nanoparticles into plastic crystals and uniform metacrystals. *Angew. Chem. Int. Ed. Engl.* **59**, 21183–21189 (2020).

23. Y. Wang, J. Chen, R. Li, A. Gotz, D. Drobek, T. Przybilla, S. Hubner, P. Pelz, L. Yang, B. Apeleo Zubiri, E. Spiecker, M. Engel, X. Ye, Controlled self-assembly of gold nanotetrahedra into quasicrystals and complex periodic supracrystals. *J. Am. Chem. Soc.* **145**, 17902–17911 (2023).

24. P. F. Damasceno, M. Engel, S. C. Glotzer, Crystalline assemblies and densest packings of a family of truncated tetrahedra and the role of directional entropic forces. *ACS Nano* **6**, 609–614 (2012).

25. A. P. Gantapara, J. de Graaf, R. van Roij, M. Dijkstra, Phase diagram and structural diversity of a family of truncated cubes: Degenerate close-packed structures and vacancy-rich states. *Phys. Rev. Lett.* **111**, 015501 (2013).

26. P. F. Damasceno, M. Engel, C. Glotzer Sharon, Predictive self-assembly of polyhedra into complex structures. *Science* **337**, 453–457 (2012).

27. S. Torquato, Y. Jiao, Dense packings of the platonic and archimedean solids. *Nature* **460**, 876–879 (2009).

28. U. Agarwal, F. A. Escobedo, Mesophase behaviour of polyhedral particles. *Nat. Mater.* **10**, 230–235 (2011).

29. B. W. Goodfellow, Y. Yu, C. A. Bosoy, D. M. Smilgies, B. A. Korgel, The role of ligand packing frustration in body-centered cubic (bcc) superlattices of colloidal nanocrystals. *J. Phys. Chem. Lett.* **6**, 2406–2412 (2015).

30. R. Li, J. Zhang, R. Tan, F. Gerdes, Z. Luo, H. Xu, J. A. Hollingsworth, C. Klinke, O. Chen, Z. Wang, Competing interactions between various entropic forces toward assembly of Pt_3Ni octahedra into a body-centered cubic superlattice. *Nano Lett.* **16**, 2792–2799 (2016).

31. M. A. Boles, D. V. Talapin, Self-assembly of tetrahedral CdSe nanocrystals: Effective "patchiness" via anisotropic steric interaction. *J. Am. Chem. Soc.* **136**, 5868–5871 (2014).

32. W. Ding, Y. Xia, H. Song, T. Li, D. Yang, A. Dong, Macroscopic superlattice membranes self-assembled from gold nanobipyramids with precisely tunable tip arrangements for SERS. *Angew. Chem. Int. Ed. Engl.* **63**, e202401945 (2024).

33. B. Gao, G. Arya, A. R. Tao, Self-orienting nanocubes for the assembly of plasmonic nanojunctions. *Nat. Nanotechnol.* **7**, 433–437 (2012).

34. Y. Zhang, D. D. Xu, I. Tanriover, W. Zhou, Y. Li, R. López-Arteaga, K. Aydin, C. A. Mirkin, Nonlinear optical colloidal metacrystals. *Nat. Photonics* **19**, 20–27 (2025).

35. M. R. Jones, K. D. Osberg, R. J. Macfarlane, M. R. Langille, C. A. Mirkin, Templated techniques for the synthesis and assembly of plasmonic nanostructures. *Chem. Rev.* **111**, 3736–3827 (2011).

36. F. Schulz, O. Pavelka, F. Lehmkühler, F. Westermeier, Y. Okamura, N. S. Mueller, S. Reich, H. Lange, Structural order in plasmonic superlattices. *Nat. Commun.* **11**, 3821 (2020).

37. M. Herran, S. Juergensen, M. Kessens, D. Hoeing, A. Köppen, A. Sousa-Castillo, W. J. Parak, H. Lange, S. Reich, F. Schulz, E. Cortés, Plasmonic bimetallic two-dimensional supercrystals for H_2 generation. *Nat. Catal.* **6**, 1205–1214 (2023).

38. J. Gong, R. S. Newman, M. Engel, M. Zhao, F. Bian, S. C. Glotzer, Z. Tang, Shape-dependent ordering of gold nanocrystals into large-scale superlattices. *Nat. Commun.* **8**, 14038 (2017).

39. C.-W. Liao, Y.-S. Lin, K. Chanda, Y.-F. Song, M. H. Huang, Formation of diverse supercrystals from self-assembly of a variety of polyhedral gold nanocrystals. *J. Am. Chem. Soc.* **135**, 2684–2693 (2013).

40. Y. H. Lee, C. L. Lay, W. Shi, H. K. Lee, Y. Yang, S. Li, X. Y. Ling, Creating two self-assembly micro-environments to achieve supercrystals with dual structures using polyhedral nanoparticles. *Nat. Commun.* **9**, 2769 (2018).

41. X. Huang, J. Zhu, B. Ge, F. Gerdes, C. Klinke, Z. Wang, In situ constructing the kinetic roadmap of octahedral nanocrystal assembly toward controlled superlattice fabrication. *J. Am. Chem. Soc.* **143**, 4234–4243 (2021).

42. J. Zhang, Z. Luo, B. Martens, Z. Quan, A. Kumbhar, N. Porter, Y. Wang, D. M. Smilgies, J. Fang, Reversible Kirkwood-Alder transition observed in Pt_3Cu_2 nanoctahedron assemblies under controlled solvent annealing/drying conditions. *J. Am. Chem. Soc.* **134**, 14043–14049 (2012).

43. J. Zhang, Z. Luo, Z. Quan, Y. Wang, A. Kumbhar, D. M. Smilgies, J. Fang, Low packing density self-assembled superstructure of octahedral Pt_3Ni nanocrystals. *Nano Lett.* **11**, 2912–2918 (2011).

44. C. A. Mirkin, S. H. Petrosko, Inspired beyond nature: Three decades of spherical nucleic acids and colloidal crystal engineering with DNA. *ACS Nano* **17**, 16291–16307 (2023).

45. C. Yi, H. Liu, S. Zhang, Y. Yang, Y. Zhang, Z. Lu, E. Kumacheva, Z. Nie, Self-limiting directional nanoparticle bonding governed by reaction stoichiometry. *Science* **369**, 1369–1374 (2020).

46. J. Xia, N. Horst, H. Guo, A. Travesset, Superlattices of nanocrystals with polystyrene ligands: From the colloidal to polymer limit. *Macromolecules* **52**, 8056–8066 (2019).

47. J. Chen, A. Fasoli, J. D. Cushen, L. Wan, R. Ruiz, Self-assembly and directed assembly of polymer grafted nanocrystals via solvent annealing. *Macromolecules* **50**, 9636–9646 (2017).

48. J. Zhang, P. J. Santos, P. A. Gabrys, S. Lee, C. Liu, R. J. Macfarlane, Self-assembling nanocomposite tectons. *J. Am. Chem. Soc.* **138**, 16228–16231 (2016).

49. J. Choi, H. Dong, K. Matyjaszewski, M. R. Bockstaller, Flexible particle array structures by controlling polymer graft architecture. *J. Am. Chem. Soc.* **132**, 12537–12539 (2010).

50. S. K. Kumar, N. Jouault, B. Benicewicz, T. Neely, Nanocomposites with polymer grafted nanoparticles. *Macromolecules* **46**, 3199–3214 (2013).

51. R. M. Choueiri, E. Galati, H. Therien-Aubin, A. Klinkova, E. M. Larin, A. Querejeta-Fernandez, L. Han, H. L. Xin, O. Gang, E. B. Zhulina, M. Rubinstein, E. Kumacheva, Surface patterning of nanoparticles with polymer patches. *Nature* **538**, 79–83 (2016).

52. A. J. Chancellor, B. T. Seymour, B. Zhao, Characterizing polymer-grafted nanoparticles: From basic defining parameters to behavior in solvents and self-assembled structures. *Anal. Chem.* **91**, 6391–6402 (2019).

53. C. Yi, Y. Yang, B. Liu, J. He, Z. Nie, Polymer-guided assembly of inorganic nanoparticles. *Chem. Soc. Rev.* **49**, 465–508 (2020).

54. H. Yun, J. W. Yu, Y. J. Lee, J.-S. Kim, C. H. Park, C. Nam, J. Han, T.-Y. Heo, S.-H. Choi, D. C. Lee, W. B. Lee, G. E. Stein, B. J. Kim, Symmetry transitions of polymer-grafted nanoparticles: Grafting density effect. *Chem. Mater.* **31**, 5264–5273 (2019).

55. H. Yun, Y. J. Lee, M. Xu, D. C. Lee, G. E. Stein, B. J. Kim, Softness- and size-dependent packing symmetries of polymer-grafted nanoparticles. *ACS Nano* **14**, 9644–9651 (2020).

56. S. M. Taheri, S. Fischer, M. Trebbin, S. With, J. H. Schröder, J. Perlich, S. V. Roth, S. Förster, Lyotropic phase behavior of polymer-coated iron oxide nanoparticles. *Soft Matter* **8**, 12124–12131 (2012).

57. W. Zhou, Y. Li, K. Je, T. Vo, H. Lin, B. E. Partridge, Z. Huang, S. C. Glotzer, C. A. Mirkin, Space-tiled colloidal crystals from DNA-forced shape-complementary polyhedra pairing. *Science* **383**, 312–319 (2024).

58. M. Yin, S. O'Brien, Synthesis of monodisperse nanocrystals of manganese oxides. *J. Am. Chem. Soc.* **125**, 10180–10181 (2003).

59. Z. L. Wang, J. S. Yin, Y. D. Jiang, EELS analysis of cation valence states and oxygen vacancies in magnetic oxides. *Micron* **31**, 571–580 (2000).

60. Y. Chu, L. Guo, B. Xi, Z. Feng, F. Wu, Y. Lin, J. Liu, D. Sun, J. Feng, Y. Qian, S. Xiong, Embedding $MnO@Mn_3O_4$ nanoparticles in an N-doped-carbon framework derived from Mn-organic clusters for efficient lithium storage. *Adv. Mater.* **30**, 1704244 (2018).

61. W. Jakubowski, B. Kirci-Denizli, R. R. Gil, K. Matyjaszewski, Polystyrene with improved chain-end functionality and higher molecular weight by ARGET ATRP. *Macromol. Chem. Phys.* **209**, 32–39 (2008).

62. A. Nelson, Y. Zong, K. E. Fritz, J. Suntivich, R. D. Robinson, Assessment of soft ligand removal strategies: Alkylation as a promising alternative to high-temperature treatments for colloidal nanoparticle surfaces. *ACS Mater. Lett.* **1**, 177–184 (2019).

63. M. Daoud, J. P. Cotton, Star shaped polymers : A model for the conformation and its concentration dependence. *J. Phys. Fr.* **43**, 531–538 (1982).

64. A. Kim, T. Vo, H. An, P. Banerjee, L. Yao, S. Zhou, C. Kim, D. J. Milliron, S. C. Glotzer, Q. Chen, Symmetry-breaking in patch formation on triangular gold nanoparticles by asymmetric polymer grafting. *Nat. Commun.* **13**, 6774 (2022).

65. K. C. Elbert, W. Zygmunt, T. Vo, C. M. Vara, D. J. Rosen, N. M. Krook, S. C. Glotzer, C. B. Murray, Anisotropic nanocrystal shape and ligand design for co-assembly. *Sci. Adv.* **7**, eabf9402 (2021).

66. K. C. Elbert, T. Vo, N. M. Krook, W. Zygmunt, J. Park, K. G. Yager, R. J. Composto, S. C. Glotzer, C. B. Murray, Dendrimer ligand directed nanoplate assembly. *ACS Nano* **13**, 14241–14251 (2019).

67. N. Metropolis, A. W. Rosenbluth, M. N. Rosenbluth, A. H. Teller, E. Teller, Equation of state calculations by fast computing machines. *J. Chem. Phys.* **21**, 1087–1092 (1953).

68. P. G. de Gennes, Polymers at an interface; a simplified view. *Adv. Colloid Interface Sci.* **27**, 189–209 (1987).

69. Z.-G. Wang, 50th anniversary perspective: Polymer conformation—A pedagogical review. *Macromolecules* **50**, 9073–9114 (2017).

70. A. Dong, J. Chen, P. M. Vora, J. M. Kikkawa, C. B. Murray, Binary nanocrystal superlattice membranes self-assembled at the liquid–air interface. *Nature* **466**, 474–477 (2010).

71. Y. Tian, J. R. Lhermitte, L. Bai, T. Vo, H. L. Xin, H. Li, R. Li, M. Fukuto, K. G. Yager, J. S. Kahn, Y. Xiong, B. Minevich, S. K. Kumar, O. Gang, Ordered three-dimensional nanomaterials using DNA-prescribed and valence-controlled material voxels. *Nat. Mater.* **19**, 789–796 (2020).

72. T. Vo, S. C. Glotzer, A theory of entropic bonding. *Proc. Natl. Acad. Sci. U.S.A.* **119**, e2116414119 (2022).

73. T. Vo, Entropic bonding—Not quite so simple behaviors from simple hard particles. *Annu. Rev. Chem. Biomol. Eng.* **16**, 147 (2025).

74. H. Minkowski, Dichteste gitterförmige lagerung kongruenter körper. *Nachr. Akad. Wiss. Göttingen Math. Phys.* , 311–355 (1904).

75. D. K. Smith, B. Goodfellow, D.-M. Smilgies, B. A. Korgel, Self-assembled simple hexagonal AB_2 binary nanocrystal superlattices: SEM, GISAXS, and defects. *J. Am. Chem. Soc.* **131**, 3281–3290 (2009).

76. J. K. Bosworth, M. Y. Paik, R. Ruiz, E. L. Schwartz, J. Q. Huang, A. W. Ko, D.-M. Smilgies, C. T. Black, C. K. Ober, Control of self-assembly of lithographically patternable block copolymer films. *ACS Nano* **2**, 1396–1402 (2008).

77. T. Hanrath, J. J. Choi, D.-M. Smilgies, Structure/processing relationships of highly ordered lead salt nanocrystal superlattices. *ACS Nano* **3**, 2975–2988 (2009).

78. G. van Anders, N. K. Ahmed, R. Smith, M. Engel, S. C. Glotzer, Entropically patchy particles: Engineering valence through shape entropy. *ACS Nano* **8**, 931–940 (2014).

79. A. H. Reidies, "Manganese compounds" in *Ullmann's Encyclopedia of Industrial Chemistry* (Wiley-VCH GmbH, Weinheim, Germany, 2000).

80. P. A. van Aken, B. Liebscher, Quantification of ferrous/ferric ratios in minerals: New evaluation schemes of Fe L_{23} electron energy-loss near-edge spectra. *Phys. Chem. Miner.* **29**, 188–200 (2002).

81. T. D. Schladt, T. Graf, W. Tremel, Synthesis and characterization of monodisperse manganese oxide nanoparticles-evaluation of the nucleation and growth mechanism. *Chem. Mater.* **21**, 3183–3190 (2009).
